# Assistance force-line of exosuit affects ankle multidimensional motion: a theoretical and experimental study

**DOI:** 10.1186/s12984-024-01386-x

**Published:** 2024-05-28

**Authors:** Xinyue Zhang, Ying Li, Ronglei Sun

**Affiliations:** Institute of Medical Equipment Science and Engineering, State Key Laboratory of Intelligent Manufacturing Equipment and Technology, School of Mechanical Science and Engineering, Huazhong University of Science and Technology, Wuhan, China

**Keywords:** Ankle exosuit, Assistance force line, Plantarflexion, Inversion, Subtalar joint, Talocrural joint

## Abstract

**Background:**

The talocrural joint and the subtalar joint are the two major joints of the ankle-joint complex. The position and direction of the exosuit force line relative to these two joint axes can influence ankle motion. We aimed to understand the effects of different force-lines on ankle multidimensional motion.

**Methods:**

In this article, three assistance force line schemes for ankle exosuits were proposed: perpendicular to the talocrural joint axis (PT), intersecting with the subtalar joint axis (IS), and parallel to the triceps surae (PTS). A theoretical model was proposed to calculate the exosuit’s assistance moment. Seven participants completed four experimental tests of ankle plantarflexion, including three passive motions assisted by the PT, PTS and IS schemes, and one active motion without exosuit assistance (Active).

**Results:**

The simulation results demonstrated that all three exosuits were able to produce significant moments of ankle plantarflexion. Among these, the PT scheme exhibited the highest moments in all dimensions, followed by the PTS and IS schemes. The experimental findings confirmed the effectiveness of all three exosuit schemes in assisting ankle plantarflexion. Additionally, as the assistive force lines approached the subtalar joint, there was a decrease in ankle motion assisted by the exosuits in non-plantarflexion directions, along with a reduction in the average distance of ankle angle curves relative to active ankle motion. Furthermore, the linear correlation coefficients between inversion and plantarflexion, adduction and plantarflexion, and adduction and inversion gradually converged toward active ankle plantarflexion motion.

**Conclusions:**

Our research indicates that the position of the exosuit force line to the subtalar joint has a significant impact on ankle inversion and adduction. Among all three schemes, the IS, which has the closest distance to the subtalar joint axes, has the greatest kinematic similarity to active ankle plantarflexion and might be a better choice for ankle assistance and rehabilitation.

**Supplementary Information:**

The online version contains supplementary material available at 10.1186/s12984-024-01386-x.

## Background

The ankle plays an important role in generating power and propelling the body forward during movement [1–3]. Ankle exosuits are wearable devices that are designed to enhance human movement performance or aid in ankle rehabilitation [4–7], primarily by assisting ankle plantarflexion motion [8, 9]. To evaluate the effects of these exosuits on ankle function and human gait, a variety of metrics have been proposed. Gait parameters and ankle joint angles are commonly used to assess the impact of exosuits on the ankle and human kinematics [10, 11]. Additionally, ankle joint moment, power, and metabolic power are utilized to evaluate their effects on ankle energy consumption and gait metabolic cost [12–15]. Furthermore, current ankle exosuits research primarily focuses on the sagittal plane motion of the ankle joint, with little attention given to the effects on inversion-eversion and abduction-adduction motion during assisting plantarflexion.

**Fig. 1:**
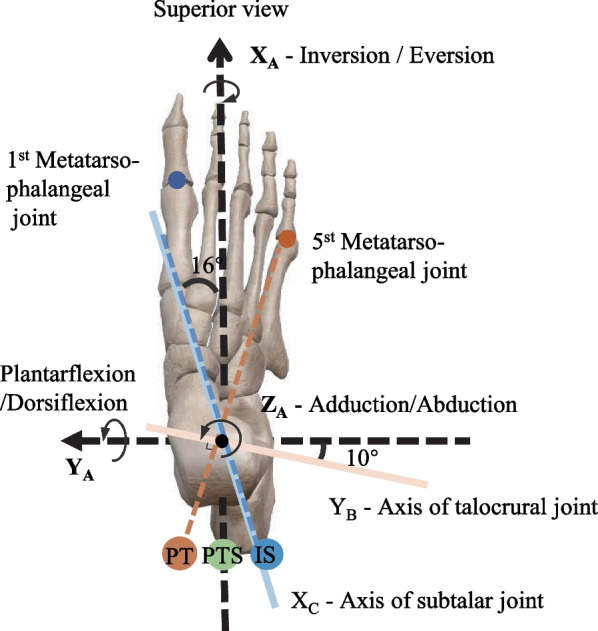
The ankle joint axes and three assistance force line schemes. The ankle rotation was described using the coordinate system $\{A\}$. Rotation around the $X_{\textrm{A}}$-axis was defined as inversion/eversion, rotation around the $Y_{\textrm{A}}$-axis was defined as plantarflexion/dorsiflexion, and rotation around the $Z_{\textrm{A}}$-axis was defined as adduction/abduction. The subtalar joint’s axis, represented by a solid blue line, is inclined at 16 degrees from the $X_{\textrm{A}}$-axis, while the talocrural joint’s axis, depicted by a solid orange line, is tilted at 10 degrees from the $Y_{\textrm{A}}$-axis. The drive-cable arrangements for the three force line schemes are indicated by the dashed lines. In the PT scheme, the drive cable runs parallel to the calf, then passes the medial side of the heel (orange dot), turns towards the fifth metatarsophalangeal joint along the orange dashed line in the sole, and ultimately fixed to the dorsal surface of the foot. In the PTS scheme, the drive cable runs parallel to the calf and is fixed in the center of the heel (green dot). In the IS scheme, the drive cable is parallel to the calf and directed towards the lateral side of the heel (blue dot), then turns towards the first metatarsophalangeal joint along the blue dashed line in the sole, and ultimately fixed to the dorsal surface of the foot

Considering the musculoskeletal anatomy of the ankle-joint complex and the structure of ankle exosuits, ankle non-plantarflexion motion (i.e., ankle eversion/inversion and adduction/abduction) cannot be ignored. As shown in Fig. 1, ankle motion is described in three orthogonal planes: plantar/dorsiflexion along the sagittal plane, inversion/eversion along the frontal plane, and adduction/abduction along the horizontal plane [1, 16, 17]. However, the major rotation joints of the ankle-joint complex are the talocrural and subtalar joints, and their two joint axes are not parallel to the three standard three orthogonal planes [18–20]. Consequently, ankle plantarflexion is not a purely unidirectional motion but is accompanied by a three-dimensional coupling movement [16, 19]. Furthermore, the contraction structures of current exosuits, such as pneumatic muscles or Bowden lines [21], pass through the subtalar joints. Since the subtalar joint is responsible for ankle version/inversion and adduction/abduction [16, 20, 22], ankle exosuits inevitably affect the non-plantarflexion motion of the ankle.

During walking, changes in ankle inversion/eversion moment are applied to counteract deviations in side-to-side center-of-mass acceleration, ensuring balance [23, 24]. An unexpected ankle moment introduced by an exosuit may disrupt the natural ankle movements, resulting in elevated joint loads and biomechanical alterations [25–27]. These disruptions may increase susceptibility to ankle sprains [28–30]. This can be particularly problematic for those who depend on the exosuit to compensate for impaired balance or mobility [31], as it can increase the fear of falling and the incidence of falls [32–34]. Therefore, it is essential to consider the impact of the exosuit on the ankle’s natural motion and develop an approach that aligns with multidimensional movement patterns to promote safety.

**Table 1:** Typical configuration of the drive cable on the foot in cable-drive ankle exosuits

No.	References	DOF^a^	Drive-cable Pos^b^	Functionality	Target Population
1	[4]	1	$x^{-}$	Assist plantarflexion	Normal
2	[8]	1	$x^{-}$	Assist plantarflexion	Normal
3	[35]	1	$x^{+}$	Resist plantarflexion	Rehabilitation
4	[36]	2	$x^{-}, y^{-}$	Assist plantarflexion and eversion	Rehabilitation
5	[37]	2	$x^{-}, x^{+}$	Assist plantar/dorsiflexion	Rehabilitation
6	[38]	2	$x^{-}, x^{+}$	Assist plantar/dorsiflexion	Stroke patients
7	[39]	4	$x^{-}, x^{+}, y^{-}, y^{+}$	Assist plantar/dorsiflexion and inversion/eversion	Stroke patients

^a^DOF, which stands for degrees of freedom, is directly related to the number of cable drives in the exosuits

^b^ Pos: The position of the ankle exosuit’s drive cable on the foot. With the ankle joint rotation center as the reference point, $x^{+}, x^{-}, y^{+}, y^{-}$ were used to represent the four positions in which the ends of the driving cable are anchored: front, back, left, and right relative to the ankle joint, respectively

The effect of an ankle exosuit on multidimensional ankle motion depends on the relationship between the force line and the two ankle axes. In this study, we primarily used the force line to describe the position and direction of the assistive force applied to the talocrural and subtalar joint axes. Several studies have recognized the importance of the position of assistance line force and have employed double or multiple drive cables to assist multidimensional ankle motion (Table 1). These drive cables are positioned in the anterior-posterior position of the ankle joint for ankle plantar/dorsiflexion and the medial-lateral position for inversion/eversion [37, 39, 40], particularly in patients with cerebral palsy and stroke [41]. However, the current study has not sufficiently investigated the effect of the force line on multidimensional ankle motion in the one-cable-drive exosuit designed to assist ankle plantarflexion.

Our study aimed to evaluate the effects of the exosuit force line on ankle kinematics. To achieve this, we developed three force line schemes for ankle exosuits, based on the musculoskeletal anatomy and muscle distribution of the ankle (Fig. 1). The first scheme, PT, was designed to be perpendicular to the talocrural joint axis, while the second scheme, IS, was designed to intersect with the subtalar joint axis. The third scheme, PTS, was designed to be parallel to the triceps surae, a common design used in most existing exosuits. We hypothesized that the IS scheme had the least impact on the subtalar joint and the greatest kinematic similarity to active ankle plantarflexion compared to the other two schemes. To test this hypothesis, we developed a theoretical model to calculate the exosuit’s assistance moment. By comparing ankle assistance moments with and without considering the ankle joint axes, we highlighted the importance of incorporating the physiological ankle axis in exosuit analysis. To evaluate the effects of the exosuit force line on the ankle, we conducted four experimental tests, including three ankle passive motions assisted by the PT, PTS, and IS schemes, and one ankle active motion without exosuit assistance. We compared the peak angle values in three orthogonal directions, the linear correlation coefficients between the inversion angle and plantarflexion angle, between the adduction angle and plantarflexion angle, and between the adduction angle and inversion angle, and the distance of ankle motion between exosuit-assisted and active ankle motion to evaluate the extent to which each exosuit scheme resembled ankle active plantarflexion.

**Fig. 2:**
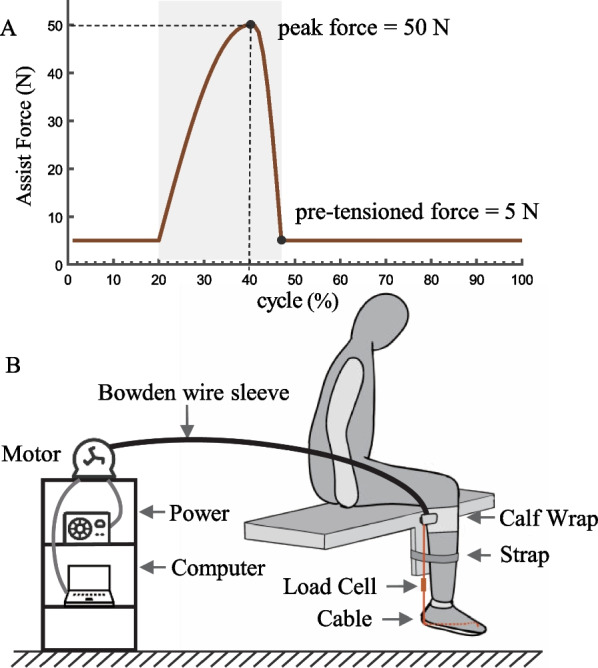
The ankle exosuit experimental platform.** A** The assist force curve. ** B** The ankle exosuit system and experimental protocol. The participant was seated on a platform with the ankle exosuit on the right leg. Assisting force was transmitted to the ankle via Bowden cables, actuated by a motor positioned on the side shelf. The calf was fastened to the platform with straps to prevent knee joint rotation during the experiment

## Methods

### Ankle exosuit structure design

**Fig. 3:**
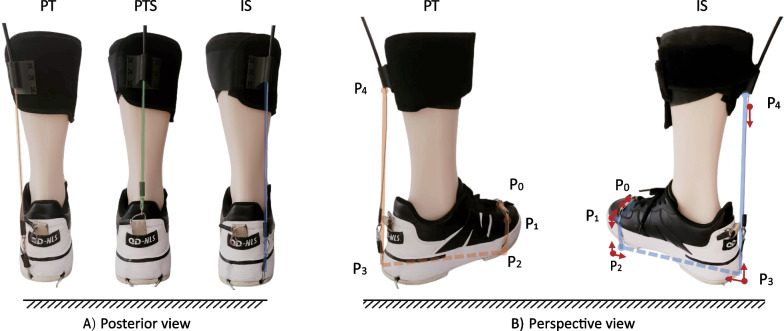
The multi-dimensional views of three ankle exosuits.** A** The posterior view of the PT, PTS, and IS ankle exosuits, with the drive cable on the medial, middle, and lateral aspects of the right leg, respectively.** B** The perspective view of the IS and PT exosuits, including the points and direction of equivalent forces application. The cable arrangement is depicted as a solid and dashed line, representing the visible and invisible parts, respectively. The red arrows signify the direction of the force for the given points

We developed three force line schemes for ankle exosuits, based on the musculoskeletal anatomy and muscle distribution of the ankle. The Bowden cables originate from the motor reel and are routed through the Bowden cable housing before arriving at the calf wrap located on the posterior aspect of the lower leg. From there, the cables run parallel to the calf and extend to the heel (Fig. 2B), while the assist force was measured using a load cell series connection with the cable. However, the positioning of the cables on the heel and foot sole differs among the three schemes (Fig. 3).

The IS scheme is routed to pass the lateral side of the heel, at a distance of approximately 3 cm from the midpoint of the heel. To ensure durability, a Teflon hose is embedded within the sole of the shoe to house the cable. The cable then passes through this hose to reach the underside of the first metatarsophalangeal joint. Finally, the cable is securely fastened by looping it around the dorsal surface of the foot.

The PT scheme is a symmetrical arrangement with the IS scheme. After passing through the calf wrap, it follows a parallel path to the calf and extends toward the medial side of the heel, maintaining a distance of approximately 3 cm from the midpoint of the heel. At the sole, the PT’s cable intersects with the IS’s cable before continuing toward the fifth metatarsophalangeal joint. Finally, the PT cable is securely fastened to the dorsal surface of the foot.

The PTS scheme is arranged to mimic the triceps surae muscle. It runs parallel to the calf and is fixed at the center of the heel.

### Control

The Bowden cable is an elastic component with complex surface friction characteristics, which can lead to the occurrence of nonlinear phenomena such as hysteresis and backlash during control. To address this issue, a p-type iterative learning algorithm is used in our study to track the assist force. In the experiment, the error of assist force from the current cycle is measured to correct the moment of the motor output shaft in the subsequent gait cycle. The targeted assist force is reached by persistently iterating.

Based on the moment of ankle active plantarflexion, we designed the assist force to an unimodal curve (Fig. 2A). The cycle duration is set to 5 s. During the first second of the cycle, the cable is maintained in a state of tension, with a tension force of 5N. Then, the tension force increases to 50N over a duration of 0.8 s. Once the peak value is attained, the cable payout process can be completed within 0.25 s. Finally, the cable remains under tension until the end of the cycle.

### The dynamics of the ankle exosuits

#### 1) Calculate the assistance moment unconsidering the ankle joint axes

The effects of the exosuits on the ankle joint can be equivalent to the application of five forces. These forces are applied at fixed and inflection points of the cable (Fig. 3B). The first point of force application, $P_0$, represents the fixed point of the cable on the dorsal surface of the foot. The second and third points of force application, $P_1$ and $P_2$ respectively, represent the inflection points where the cable changes direction from the upper to the side surface and then from the side to the sole. The fourth point of force application, $P_3$, indicates the fixed point on the heel. The entry point of the cable on the calf wrap is marked as $P_4$. The PTS can be considered a special scheme where $P_0$, $P_1$, $P_2$, and $P_3$ coincide at the heel.

In the superior view of the foot-ankle complex, the center of ankle joint rotation serves as the origin of the coordinate system $\{A\}$, as depicted in Fig. 1. In this coordinate system, the coordinates of the five points are as follows:

1 $$\begin{aligned} P_{i 0}=\left( P_{i 0}(x), P_{i 0}(y), P_{i 0}(y)\right) , i=0,1,2,3,4 \end{aligned}$$

As the ankle motion during exosuits assistance, the coordinates of the five points will change correspondingly. Assuming that the ankle joint rotates $\theta _x$, $\theta _y$, and $\theta _z$ relative to the three axes of $\textrm{X}_{\textrm{A}}$-axis,$\textrm{Y}_{\textrm{A}}$-axis, and $\textrm{Z}_{\textrm{A}}$-axis, respectively, the coordinates of the five points after the rotation will be:

2 $$\begin{aligned} \begin{aligned} P_i&=R_z\left( \theta _z\right) R_y\left( \theta _y\right) R_x\left( \theta _x\right) P_{i 0}, i=0,1,2,3 \\ P_4&=P_{40} \end{aligned} \end{aligned}$$

where, $R_i\left( \theta _i\right)$ represents the rotation matrix that rotates around the i-axis by an angle $\theta _i$. Since the calf is fixed, there is no coordinate change in $P_4$.

Assuming that the tension *F* is equal at each point along the cable without considering friction, the forces $F_i$ acting on the ankle are:

3 $$\begin{aligned} \begin{aligned} F_0&=F \frac{\overrightarrow{P_0 P_1}}{\left| \overrightarrow{P_0 P_1}\right| } \\ F_1&=F \frac{\overrightarrow{P_1 P_0}}{\left| \overrightarrow{P_1 P_0}\right| }+F \frac{\overrightarrow{P_1 P_2}}{\left| \overrightarrow{P_1 P_2}\right| } \\ F_2&=F \frac{\overrightarrow{P_2 P_1}}{\left| \overrightarrow{P_2 P_1}\right| }+F \frac{\overrightarrow{P_2 P_3}}{\left| \overrightarrow{P_2 P_3}\right| } \\ F_3&=F \frac{\overrightarrow{P_3 P_2}}{\left| \overrightarrow{P_3 P_2}\right| }+F \frac{\overrightarrow{P_3 P_4}}{\left| \overrightarrow{P_3 P_4}\right| } \end{aligned} \end{aligned}$$

where $F_i$ is the force at $P_i$.

The assistance moment, denoted as $M_{\text{ ank1 } }$, is calculated by the sum of the forces cross multiplied by their respective moment arms about the ankle joint. This is expressed as:

4 $$\begin{aligned} M_{\text{ ank1 }}=\overrightarrow{OP_0} \times F_0+\overrightarrow{OP_1} \times F_1+\overrightarrow{OP_2} \times F_2+\overrightarrow{OP_3} \times F_3 \end{aligned}$$

where *O* is the origin of the coordinate system $\{A\}$.

#### 2) Calculate the assistance moment considering the ankle joint axes

Due to the non-orthogonality of the ankle joint axes, the assistance moment of the ankle response may differ from the assistance moment input by the exosuit. To understand this difference, our study calculated the assistance moment of the ankle joint response based on the characteristics of the ankle joint axis. Specifically, we independently calculated the assist moments for the talocrural and subtalar joints and then added them together to determine the overall assist moment to the ankle.

To calculate the assistance moment on the subtalar joint, a new coordinate system $\{B\}$ was constructed by rotating the original coordinate system $\{A\}$. In this new system, the $\textrm{X}_{\textrm{B}}$-axis aligns with the axis of the subtalar joint, and the assistive moment on the subtalar joint is the moment acting on the $\textrm{X}_{\textrm{B}}$-axis (Fig. 1).

First, we solved the coordinates of the force application points in the coordinate system $\{B\}$. The subtalar joint’s rotation axis is known to form an angle of $\varphi _z=16^{\circ }$ with the sagittal plane and an angle of $\varphi _x=42^{\circ }$ with the horizontal plane. Using geometric relations, we obtained the rotation matrix and the force application point coordinates in the coordinate system $\{B\}$:

5 $$\begin{aligned} P_{\text{ isubtalar } }=R_y\left( \varphi _y\right) R_z\left( \varphi _z\right) P_i, i=0,1,2,3,4 \end{aligned}$$

where,

$$\begin{aligned} \begin{aligned} R_z\left( \varphi _z\right)&=\left[ \cos \left( \varphi _z\right) , \sin \left( \varphi _z\right) , 0;-\sin \left( \varphi _z\right) , \cos \left( \varphi _z\right) , 0; 0,0,1\right] \\ R_y\left( \varphi _y\right)&=\left[ \cos \left( \varphi _y\right) , 0,-\sin \left( \varphi _y\right) ; 0,1,0; \sin \left( \varphi _y\right) , 0, \cos \left( \varphi _y\right) \right] \\ \cos \left( \varphi _y\right)&=\frac{\sqrt{\tan ^2\left( \varphi _z\right) +1}}{\sqrt{\tan ^2\left( \varphi _z\right) +\tan ^2\left( \varphi _x\right) +1}} \\ \sin \left( \varphi _y\right)&=-\frac{\tan \left( \varphi _x\right) }{\sqrt{\tan ^2\left( \varphi _z\right) +\tan ^2\left( \varphi _x\right) +1}} \end{aligned} \end{aligned}$$

Then, we substituted the coordinates of $P_{\text {isubtalar}}$ into Eq.(3, 4) to solve the subtalar joint moment $M_{\text {subtalar}}^B$ under the coordinate system $\{B\}$.

Finally, we projected the subtalar joint moment onto the coordinate system $\{A\}$:

6 $$\begin{aligned} M_{\text{ subtalar} }^A=R_z\left( \varphi _z\right) ^{\prime } R_y\left( \varphi _y\right) ^{\prime } M_{\text{ subtalar}}^B[1,0,0]^{\prime } \end{aligned}$$

where, $R_z\left( \varphi _z\right) ^{\prime }$ and $R_y\left( \varphi _y\right) ^{\prime }$ are the inverses of $R_z\left( \varphi _z\right)$ and $R_y\left( \varphi _y\right)$.

Similarly, we calculated the moment on the talocrural joint by rotating the original coordinate system $\{A\}$ to the coordinate system $\{C\}$, where the $\textrm{Y}_{\textrm{C}}$-axis aligns with the talocrural joint axis. By determining the exosuit moment on the $\textrm{Y}_{\textrm{C}}$-axis in the coordinate system $\{C\}$, we calculated the talocrural joint moment.

To begin, we solved for the coordinates of the force application points in the coordinate system $\{C\}$. The rotation axis of the talocrural joint forms an angle of $\beta _x=6^{\circ }$ with the frontal plane, and forms an angle of $\beta _y=10^{\circ }$ with the horizontal plane. Using geometric relations, we obtained the rotation matrix and the point coordinates after rotation in the coordinate system $\{C\}$:

7 $$\begin{aligned} P_{\text{ italocrural } }=R_z\left( \beta _z\right) R_x\left( \beta _x\right) P_i, i=0,1,2,3,4 \end{aligned}$$

where,

$$\begin{aligned} \begin{aligned} R_x\left( \beta _x\right) =&{\left[ 1,0,0; \cos \left( \beta _x\right) , \sin \left( \beta _x\right) , 0; 0,-\sin \left( \beta _x\right) , \cos \left( \beta _x\right) \right] } \\ R_z\left( \beta _z\right) =&{\left[ \cos \left( \beta _z\right) , \sin \left( \beta _z\right) , 0;-\sin \left( \beta _z\right) , \cos \left( \beta _z\right) , 0; 0,0,1\right] } \\ \cos \left( \beta _z\right) =&\frac{\sqrt{\tan ^2\left( \beta _y\right) +1}}{\sqrt{\tan ^2\left( \beta _x\right) +\tan ^2\left( \beta _y\right) +1}} \\ \sin \left( \beta _z\right) =&-\frac{\tan \left( \beta _x\right) }{\sqrt{\tan ^2\left( \beta _x\right) +\tan ^2\left( \beta _y\right) +1}} \end{aligned} \end{aligned}$$

Next, we substituted $P_{\text {italocrural}}$ into the Eq.(3, 4) to solve the moment $M_{\text {talocrural}}^C$.

Finally, we projected the talocrural joint moment onto the coordinate system $\{A\}$:

8 $$\begin{aligned} M_{\text{ talocrural }}^A=R_x\left( \beta _x\right) ^{\prime } R_z\left( \beta _z\right) ^{\prime } M_{\text{ talocrural }}^C[0,1,0]^{\prime } \end{aligned}$$

where, $R_z\left( \beta _z\right) ^{\prime }$ and $R_x\left( \beta _x\right) ^{\prime }$ are the inverses of $R_z\left( \beta _z\right)$ and $R_x\left( \beta _x\right)$.

In summary, the comprehensive moment $M_{\text {ank2}}$ of the exosuit to the ankle joint is obtained:

9 $$\begin{aligned} \begin{aligned} M_{\text{ ank2 }}&=M_{\text{ subtalar } }^A+M_{\text{ talocrural } }^A \\&=f\left( P_{30}, F, \theta _x, \theta _y, \theta _z\right) . \end{aligned} \end{aligned}$$

The detailed calculation process and results are documented in the supplementary materials. MATLAB (MathWorks Inc., USA) was used for solving the formulas.

### Simulation protocol

In our study, the simulation calculated the assistance moment curve under two conditions: one considering the ankle axes using Eq. (9) and the other without using Eq. (4). The applied assistive force was held constant at 1, and the coordinates of point $P_{3 0}$ were ($-$0.09, ± 0.03, $-$0.11). The ankle angle curves used as model inputs are presented below:

10 $$\begin{aligned} \theta _q=\theta _{\text {qmax }} \sin (t)(q=x, y, z),\quad 0<t<\pi \end{aligned}$$

where $\theta _{qmax}$ denoted the maximum ankle angle in the q-axis during the exosuit assist experiments. All results are dimensionless.

### Participants

Seven adult males completed the experiment (20.1 ± 3.1 years, 178 ± 35 cm, 72.0 ± 7.1kg). All participants provided informed consent and were informed of the possible consequences of the study. The Science and Technology Committee of Huazhong University of Science and Technology approved the experimental protocol for using human subjects.

### Experimental protocol

Participants were instructed to wear the exosuit on their right leg and sit on a table to complete ankle plantarflexion. To reduce the impact from rotation from the hip and knee joints, the strap was utilized to secure the participants’ right leg to the platform. The experiment consisted of four tests: an active ankle plantarflexion motion and three passive ankle plantarflexion motions assisted by PT, PTS, and IS, respectively. During the active test, participants were directed to actively perform ankle plantarflexion, following the operator’s commands. The exosuit was worn but not actuated, and did not impede ankle joint motion. During the three passive ankle plantarflexion tests, participants were instructed to consistently maintain a relaxed state while completing plantarflexion with the assistance of the exosuit. Each cycle lasted for five seconds, and participants repeated ankle plantarflexion movements at least 20 times during each test.

While seated, the initial ankle position was defined with the soles of the feet parallel to the ground, maintaining a 90° angle between the calf and sole. Throughout the experiment, participants were instructed to monitor the motion of their ankle joints by a mirror and make an effort to return the ankle joint to its initial position before each plantarflexion.

### Measurements and data processing

Kinematic data of the ankle in three dimensions were collected using a 7-camera reflective marker motion capture system (Vicon, Oxford Metrics, Oxford, UK; 100 Hz). Seven infrared reflective markers were placed on specific anatomical landmarks on the right leg, including the lateral knee joint $\left( P_{\text{ knee } }\right)$, medial/lateral malleolus $\left( P_{\text {ankle}\_\text {med}}/ P_{\text{ ankle }\_\text {lat }}\right)$, heel $\left( P_{\text{ heel }}\right)$, and 1st, 2nd, and 5th metatarsophalangeal joints $\left( P_{\text{ toe1 }}, P_{\text{ toe2 }}, P_{\text{ toe5 }}\right)$. The three-dimensional coordinates of each marker were filtered with a 6-Hz low pass Butterworth filter.

The three-dimensional angle of the ankle joint was calculated according to geometric relationships:

11 $$\begin{aligned} \begin{aligned} \theta _{\text{ IN } }&=\arccos \left( \frac{\overrightarrow{leg_{\Vert }} \cdot \overrightarrow{{ foot }_{\perp }}}{\left| \overrightarrow{leg_{\Vert }}\right| \left| \overrightarrow{{ foot }_{\perp }}\right| }\right) -\frac{\pi }{2} \\ \theta _{\text {AD }}&=\arccos \left( \frac{\overrightarrow{{ foot }_{\Vert }} \cdot \overrightarrow{leg_{\perp }}}{\left| \overrightarrow{{ foot }_{\Vert }}\right| \left| \overrightarrow{leg_{\perp }}\right| }\right) -\frac{\pi }{2} \\ \theta _{\text{ PF } }&=\arccos \left( \frac{\overrightarrow{{ foot }_{\Vert }} \times \overrightarrow{leg_{\perp }} \cdot \overrightarrow{leg_{\Vert }}}{\left| \overrightarrow{leg_{\Vert }}\right| \left| \overrightarrow{{foot }_{\Vert }} \times \overrightarrow{leg_{\perp }}\right| }\right) \\ \\ \overrightarrow{{ leg }_{\Vert }}&=P_{\text{ knee } }-P_{\text{ ankle }\_\text {lat }}\\ \overrightarrow{{ leg }_{\perp }}&=P_{\text{ ankle }\_\text {med } }-P_{\text{ ankle }\_\text {lat }}\\ \overrightarrow{{ Foot }_{\Vert }}&=P_{\text{ toe2 } }-P_{\text{ heel } }\\ \overrightarrow{{ Foot }_{\perp }}&=P_{\text{ toe1 } }-P_{\text{ toe5 } } \end{aligned} \end{aligned}$$

where, $\theta _{\text{ IN, } } \theta _{\text{ AD, } }$ and $\theta _{\text{ PF } }$ are the inversion, adduction, and plantarflexion angles of the ankle, respectively. The data from the last one minute was collected. The curves of ankle angle from each condition were averaged across participants.

**Table 2:** The mean (standard deviation) and statistic analysis of the ankle peak angles and the linear correlation coefficients in four experimental schemes

Valve	Active	PTS				PT				IS			
	Mean(SD)	Mean(SD)	P-value	ES	Power	Mean(SD)	P-value	ES	Power	Mean(SD)	P-value	ES	Power
$\text{ PF } \text{ peak }$	38.36(4.86)	39.59(4.15)	0.62	− 0.27	0.09	38.41(3.66)	0.99	− 0.01	0.05	42.52(4.46)^∗^	0.12	− 0.89	0.48
$\text{ IN } \text{ peak }$	6.26(3.59)	28.17(3.24)^∗^	< 0.05	− 6.41	1.00	13.83(1.46)^∗^	< 0.05	− 2.76	1.00	10.79(3.89)^∗^	<0.05	− 1.21	0.79
$\text{ AD } \text{ peak }$	8.62(4.94)	17.66(4.58)^∗^	< 0.05	− 1.90	0.98	11.66(4.33)	0.24	− 0.65	0.28	9.87(5.34)	0.66	− 0.24	0.09
$\text{ IN/PF }$	0.22(0.13)	0.78(0.16)^∗^	< 0.05	− 3.84	1.00	0.41(0.08)^∗^	< 0.05	− 1.70	1.00	0.28(0.06)	0.42	− 0.54	0.99
$\text{ AD/PF }$	0.22(0.08)	0.46(0.09)^∗^	< 0.05	− 2.72	0.89	0.28(0.06)	0.27	− 0.75	0.30	0.22(0.04)	0.97	0.03	0.88
$\text{ AD/IN }$	0.97(0.35)	1.69(0.23)^∗^	< 0.05	− 2.45	0.16	1.47(0.16)^∗^	< 0.05	− 1.84	0.05	1.28(0.31)^∗^	0.18	− 0.93	0.50

* indicates that the value in the current scheme exhibits a significant difference compared to ankle active plantarflexion (P-value < 0.05 or |ES| > 0.8)

### Statistical analysis

We utilized Dynamic Time Warping (DTW) to calculate the trajectory distance between the three exosuit-assisted motions and active motion. Statistical analysis involved Bland-Altman plots to assess differences between the three exosuit-assisted motions and active motion. Additionally, statistical analyses were conducted on the linear correlation coefficients of angles in pairwise orthogonal directions and peak values of ankle angles in three directions. The normal distribution assumption was assessed using the Jarque-Bera two-sided goodness test. A one-way repeated-measures analysis of variance (ANOVA) was employed to evaluate differences between active and passive ankle plantarflexion, excluding outliers with a Thompson Tau test. The significance level for all analyses was set at $P<0.05$. We also conducted a power analysis to determine the probability of a Type II error. Our acceptance criteria for power levels ranged from 0.80 or higher. Effect sizes (ES) were calculated using Cohen’s d, which is defined as the difference of group means divided by the pooled standard deviation. This allowed us to quantify the difference between active and passive ankle plantarflexion, with an effect size of 0.2 considered small, 0.5 medium, and 0.8 large. All statistical analyses were performed using MATLAB (MathWorks Inc., USA).

**Fig. 4:**
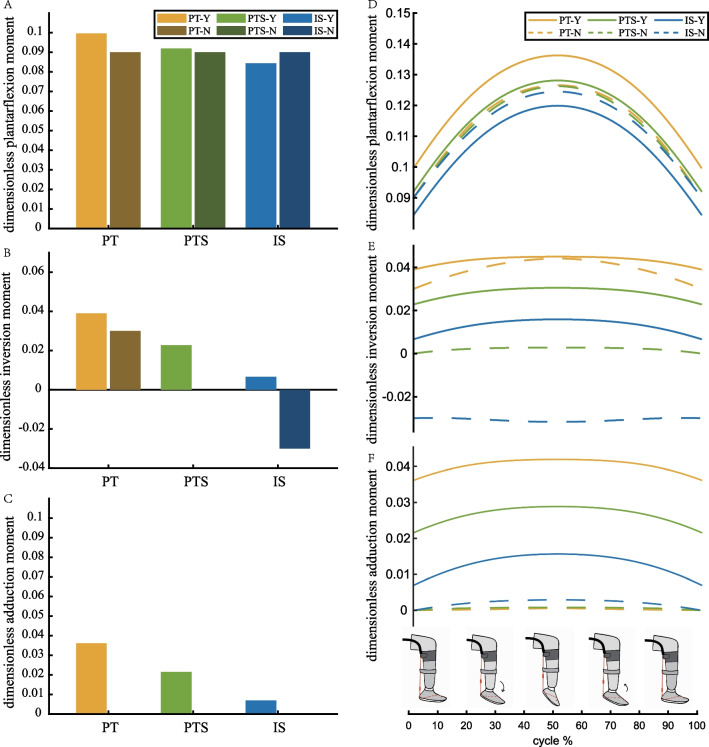
The assistance moments of the three exosuits. A-C) The non-dimensional assistance moments of plantarflexion, inversion, and adduction with the ankle maintain a plantarflexion angle of 0^∘^, respectively. D-F) The non-dimensional assistance moments curves of plantarflexion, inversion, and adduction with ankle motion, respectively. The notation “-Y” indicates that the ankle axes were considered in the analysis, while “-N” indicates that they were neglected

## Results

The ankle moments generated by the PT, PTS, and IS exosuits significantly differ when considering the non-orthogonal characteristics of ankle joint axes compared to not considering them, assuming the ankle joint maintains its initial position. Without considering the ankle joint axes, the moments for plantarflexion all are 0.090, inversion are 0.030 (PT), 0.000 (PTS), and $-$0.030 (IS), with all zero adduction moments. When considering the ankle joint axes, the ankle moments change as follows: plantarflexion moments of 0.0996 (PT), 0.0920 (PTS), and 0.0840 (IS), inversion moments of 0.039 (PT), 0.023 (PTS), and 0.007 (IS), and adduction moments of 0.036 (PT), 0.022 (PTS), and 0.007 (IS) (Fig. 4A−C).

Considering the changes in ankle joint angle during assistance, this study calculated the curve of assistance moments with ankle movement (Fig. 4D–F). The simulation results reveal that as the ankle angle increased, the plantarflexion, inversion, and adduction moments provided by the exosuits increased in all three directions. Additionally, all simulation results for the moment curve displayed left-right symmetry, signifying that the magnitude of the exosuit assistive moment remained unaffected by the direction of ankle motion velocity.

**Fig. 5:**
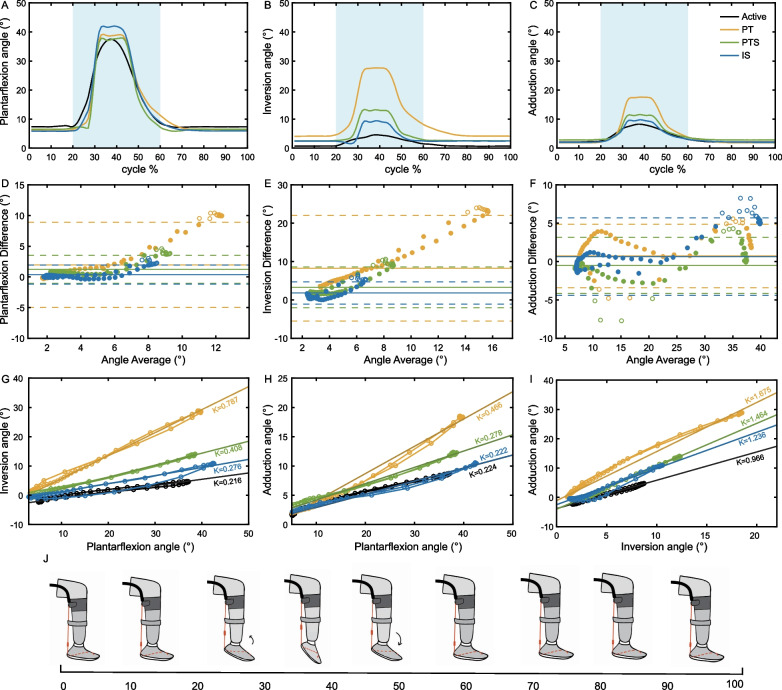
Ankle motion analysis.** A**–**C** show the average angle across a motion cycle of the ankle in the directions of plantarflexion, inversion, and adduction, respectively.** D**–**F** depict the motion differences between three exosuit-assisted and active ankle motions in the directions of plantarflexion, inversion, and adduction, utilizing Bland-Altman plots. In these plots, circles represent the angle differences of the three assistive motions relative to active ankle motion. Solid markers indicate differences within the limits, while hollow markers indicate differences beyond the limits. The horizontal dashed lines represent the limits of agreement, corresponding to the mean angle difference ± 1.96 standard deviations of angle differences, while the solid horizontal lines represent the mean angle difference.** G**−**I** illustrate the phase trajectory of ankle motion between the inversion angle and plantarflexion angle, between the adduction angle and plantarflexion angle, and between the adduction angle and inversion angle, respectively. The K value denotes the trajectory^′^s slope, representing the linear correlation coefficient between the two angles.** J** presents a schematic representation of ankle motion in a motion cycle. Each color corresponds to a specific experiment condition: black for active plantarflexion without exosuit assistance (Active), blue for IS assistance, green for PTS assistance, and yellow for PT assistance. The light blue rectangle represents the primary region of ankle motion

We assessed the differences in ankle kinematics between the three exosuit-assisted motions and active motion through motion acquisition experiments (Fig. 5A–C). During the ankle active plantarflexion experiment, the peak angles for plantarflexion, inversion, and adduction were measured at 38.36±4.86 degrees, 6.26±3.59 degrees, and 8.62±4.94 degrees, respectively. In the ankle passive plantarflexion experiment, three different exosuits, PT, PTS, and IS, were utilized. The plantarflexion peak angles for the ankle assisted by these three exosuits were found to be 39.59±4.15 degrees, 38.41 ± 3.66 degrees, and 42.5 ± 4.46 degrees, respectively. The inversion peak angles were 28.17 ± 3.26 degrees, 13.83±1.46 degrees, and 10.79±3.89 degrees, while the adduction peak angles were 17.66±4.58 degrees, 11.66 ± 4.33 degrees, and 9.87±5.34 degrees, respectively (Fig. 5D–F; Table 2).

Using Bland-Altman plots, we observed that the three assistive motions show agreement across most motion ranges with active ankle motion. However, there is a clustering of outlier points before and after reaching the peak angle (Fig. 5D–F).

In the plots of the phase trajectory diagram, it was observed that both active ankle motion and exosuit-assisted motion demonstrate an increase in ankle inversion and adduction angles as the ankle plantarflexion angle increases and shows a clear linear correlation (Fig. 5G–I). During the active ankle plantarflexion experiment, the linear correlation coefficients between the inversion angle and plantarflexion angle, between adduction angle and plantarflexion angle, and between adduction angle and inversion angle were 0.22 ± 0.13, 0.22 ± 0.08, and 0.97 ± 0.35, respectively. In the passive ankle plantarflexion experiment, the linear correlation coefficients between the inversion angle and plantarflexion angle for the three exosuits PT, PTS, and IS were 0.78 ± 0.16, 0.41 ± 0.08, and 0.28 ± 0.06, respectively. The linear correlation coefficients between the adduction angle and plantarflexion angle were 0.46 ± 0.09, 0.28 ± 0.06, and 0.22 ± 0.04, respectively. The linear correlation coefficients between the adduction angle and inversion angle were 1.69 ± 0.23, 1.47 ± 0.16, and 1.28 ± 0.31, respectively (Table 2).

When comparing the three schemes, the linear correlation coefficients of the IS scheme were found to be the closest to those of active ankle motion. No significant differences were observed between IS-assisted and active ankle motion regarding the coefficients between the inversion angle and plantarflexion angle, as well as between the adduction angle and plantarflexion angle. Within the PTS scheme, the linear correlation coefficients between the inversion angle and plantarflexion angle, as well as between the adduction angle and inversion angle, showed a statistically significant increase compared to active ankle motion (P < 0.05, or effect size: $|d|>$ 0.80; Table 2). Similarly, the three linear correlation coefficients for the PT demonstrated a statistical increase, with larger effect sizes observed in all three directions compared to PTS.

The similarity of ankle angle curves was assessed using DTW distances. In the PT scheme, the mean DTW distances to the active ankle plantarflexion angle curve were 1.02^∘^, 7.78^∘^, and 1.65^∘^ in the plantarflexion, inversion, and adduction directions, respectively (see Table 3). The root mean square distance across the three directions totaled 4.63^∘^. In the PTS scheme, the corresponding curve distances measured 0.93^∘^, 2.62^∘^, and 0.91^∘^ in the plantarflexion, inversion, and adduction directions, respectively, with a root mean square distance of 1.69^∘^. Within the IS scheme, the curve distances were 1.55^∘^, 1.79^∘^, and 0.25^∘^ in the plantarflexion, inversion, and adduction directions, respectively, with a root mean square distance of 1.37^∘^. Overall, compared to the other two schemes, the IS scheme exhibited the smallest curve distances to active ankle plantarflexion.

**Table 3:** The mean DTW distances (^∘^) of angle curves between the three exosuit-assisted motions and active motion

Rotation	PT	PTS	IS
$\textrm{PF}(+) / \textrm{DF}(-)$	1.02	0.93	1.55
$\textrm{IN}(+) / \textrm{EN}(-)$	7.78	2.62	1.79
$\textrm{AD}(+) / \textrm{AB}(-)$	1.65	0.91	0.25
$\textrm{RMS}^a$	4.63	1.69	1.37

^a^ RMS stands for the square root of the mean distance calculated in three directions

## Discussion

In this study, three exosuits, the PT, IS, and PTS, were developed based on the ankle joint axes and muscle distribution. We investigated the impact of the position and direction of the force line on the ankle’s three-dimensional motion. This was done to identify an ankle exosuit design that can provide a more natural form of assistance.

A dynamic model was developed to calculate the exosuit assistance moment. Simulation results revealed that all three schemes generated a plantarflexion moment by positioning the force line posterior to the talocrural joint axis. Among these schemes, the PT scheme, with the longest moment arm relative to the two joint axes, yielded the highest moment for these axes. Moreover, both the PT and PTS schemes induced ankle inversion and adduction moments by influencing subtalar joint motion. The PTS scheme exhibited a lower non-plantarflexion moment due to its shorter moment arm to the subtalar joint compared to the PT scheme. Conversely, the IS scheme, intersecting with or near the subtalar joint axis, had a minimal moment arm for subtalar joint motion. Consequently, the IS scheme displayed the smallest non-plantarflexion moment compared to the other two schemes.

During the exosuit assistance, the moment arm increases with the ankle plantarflexion angle, which in turn enlarges the moment and yields a larger angle of motion. That is more obvious in the PT scheme. When plantarflexion and inversion moments are generated by the ankle exosuit, corresponding plantarflexion, and inversion motion of the ankle are induced. Then, as the ankle inversion angle increases, the exosuit’s inversion moment arm of the assistance force line also increases, leading to a further increase in the inversion moment. The lateral collateral ligaments limit ankle inversion while the triangular ligament limits ankle eversion. Given the lateral collateral ligaments are weaker than the triangular ligament and the inversion motion range is larger than eversion (23^∘^ inversion, -12^∘^ eversion) [42], ankle sprains often involve ankle inversion [43, 44]. Therefore, the ankle inversion moment provided by ankle exosuits should be carefully applied.

In previous experiments conducted by Ho Seon Choi and colleagues [36, 45], they observed that a 1-DOF powered ankle-foot orthosis, which shares structural similarity with the PTS scheme, resulted in a lateral shift of the in-shoe center of pressure during walking. Their results indicated that the 1-DOF-powered ankle-foot orthosis could generate unexpected inversion moments. These findings align with our simulation and experimental results, in which the PTS scheme generated an inversion moment at the ankle joint.

We investigated the differences in ankle kinematics resulting from three exosuit schemes through exosuit-assisted experiments, and the findings were consistent with the simulation results. All three schemes effectively assisted ankle plantarflexion. The PT scheme exhibited the largest increase in the peak angle of ankle inversion and adduction, followed by PTS and IS. This difference can be attributed to the fact that the PT scheme introduced the highest ankle non-plantarflexion moments, followed by the PTS and IS schemes. In contrast, the IS assistance produced the smallest peak angles for ankle inversion and adduction among the three schemes, along with the smallest DTW distance and the highest similarity to active plantarflexion.

We observed that the differences in ankle joint motion between three exosuit-assisted and active ankle motions mainly clustered before and after reaching the peak angle. This phenomenon can be attributed to the fact that, with assistance, the plantarflexion velocity of the ankle joint surpasses that of active motion. Consequently, the ankle joint reaches its limit position earlier with assistance compared to active motion, resulting in differences around the joint limit position. The coordination among the multidimensional movements of the ankle joint, rather than differences in velocity along individual movement directions, enables a clearer understanding of the impact of assistive force lines on natural ankle joint motion. Therefore, we proposed conducting further analysis of the differences in ankle joint motion across three directions between assisted and active motion using phase trajectories. From the phase plots, it was observed that as the force lines approached the subtalar joint axis, the linear correlation coefficients gradually converged towards the coefficients of active ankle joint motion.

Additionally, we need to clarify why we opted for a relatively large assistive force magnitude. The decision stemmed from the need for consistency and repeatability in our experiments. Participants were instructed to achieve the maximum plantarflexion angle possible during active ankle motion, ensuring consistent peak angles across multiple cycles. Simultaneously, to ensure that all participants achieved maximal ankle joint movement during exosuit-assisted motion, we set the peak assistive force at a relatively high level of 50N across multiple experimental trials. This applied force resulted in a notably higher plantarflexion velocity of the ankle joint compared to that observed during active motion.

In summary, this study indicates that the IS scheme has the least interference with non-plantarflexion movements and is closest to natural ankle joint motion compared to the other two schemes. Considering the ankle’s crucial role in maintaining lateral balance through actively generating inversion and eversion moments [16, 19], the IS exosuit provides the flexibility needed to support ankle inversion and eversion motions, making it a promising candidate for future applications.

Due to individual differences in the direction of ankle joint axes [46], the lines of assistance force produced by our exosuits may not be precisely perpendicular to the talocrural joint or intersect with the subtalar joint. For example, we assumed that the axis of the talocrural joint formed a 10^∘^ angle with respect to the sagittal plane based on prior research [47, 48]. However, some studies have shown that this angle can vary between 6^∘^ and 4^∘^ among different subjects [46, 49]. Nevertheless, these variations do not impact the validity of our research plan or primary conclusions. The three force lines always satisfy the following conditions: the PT has the largest force arm relative to the two axes, followed by the PTS, and then the IS.

We obtained the current findings from a sample consisting of male participants. Considering the differences in male and female biology and physiology [50, 51], we will investigate potential gender-related variations among three assistance schemes in future work.

## Conclusions

The talocrural and subtalar joints are the major rotation joints for the ankle joint complex. The assistance force line of the exosuit relative to these two joint axes is essential for ankle motion. In this study, we proposed three bionic force line schemes: perpendicular to the talocrural joint (PT), intersecting with the subtalar joint axis (IS), and parallel to the triceps calf (PTS). We conducted simulations and experiments to assess the impact of different force line configurations on multidimensional ankle motion. Our findings reveal that all three schemes effectively assisted ankle plantarflexion. As the assistive force lines approached the subtalar joint axis, exosuit-assisted motion in the non-plantarflexion direction decreased, the distance of ankle angle curves relative to active ankle motion decreased, and the linear correlation coefficients also gradually converged towards active ankle plantarflexion motion. In summary, the IS scheme exhibits the highest kinematic similarity to active ankle plantarflexion compared to the other two schemes, making it the optimal choice for ankle assistance and rehabilitation.

### Supplementary Information

**Additional file 1. **Ankle plantarflexion with PT exosuit assistance An illustrative video for understanding pre-experiment.

**Additional file 2. **Exosuit assistance moments - Detailed derivation process and results.

## Data Availability

The data collected and analyzed in the current study are available in the manuscript and supplementary data. More details are available from the corresponding author upon request.

## Abbreviations

PT: Perpendicular to the talocrural joint axis
IS: Intersecting with the subtalar joint axis
PTS: Parallel to the triceps surae
ES: Effect sizes
DTW: Dynamic Time Warping
IN: Inversion
PF: Plantarflexion
AD: Adduction
