# Supplementary material for: Assistance force-line of exosuit affects ankle multidimensional motion: a theoretical and experimental study
Source: J Neuroeng Rehabil. 2024 May 28;21:87. doi: 10.1186/s12984-024-01386-x (PMC11131222; doi:10.1186/s12984-024-01386-x)
Supplement: Supplementary file 2 — Additional file 2. Exosuit assistance moments - Detailed derivation process and results. [file 12984_2024_1386_MOESM2_ESM.pdf]

# Exosuit assistance moments calculation

### 1) Calculation of the assistance moment unconsidering the ankle joint axes

The impact of the cable-driven exosuit on the ankle-joint complex can be visualized as the result of five equivalent point loads, and the coordinates of these five points are as follows:

$$P_{0o} = [\,p0x, p0y, p0z]$$
$$P_{1o} = [p1x, p1y, p1z]$$
$$P_{2o} = [p2x, p2y, p2z]$$
$$P_{3o} = [p3x, p3y, p3z]$$
$$P_{4o} = [p3x, p3y, p4z]$$

As the ankle joint angle changes during the ankle assist procedure, the coordinates of the four points on the feet mentioned above will also change correspondingly. Assuming that the ankle joint rotates $inversion$、$flexion$ and$adduction$ relative to the three axes of XYZ during plantar flexion movement, the coordinates of the five points after rotation will be:

$$P_i = R(adduction)R(flexion)R(inversion)P_{io},\ i = 0,1,2,3$$
$$P_4 = P_{4o}$$

Where，

$$R(inversion) = [1,0,0;0,\cos(inversion),-\sin(inversion);0,\sin(inversion),\cos(inversion)]$$
$$R(flexion)=[\cos(flexion),0,\sin(flexion);0,1,0;-\sin(flexion),0,\cos(flexion)]$$
$$R(adduction) = [\cos(adduction),-\sin(adduction),0;\sin(adduction),\cos(adduction),0;0,0,1]$$

Assuming that the tension at each point on the cable is the same, and that tension is denoted by $F$, the four forces acting on the shoe are:

$$F_0 = F\frac{\overrightarrow{P_0P_1}}{|\overrightarrow{P_0P_1}|}$$
$$F_1 = F\frac{\overrightarrow{P_1P_0}}{|\overrightarrow{P_1P_0}|} + F\frac{\overrightarrow{P_1P_2}}{|\overrightarrow{P_1P_2}|}$$
$$F_2 = F\frac{\overrightarrow{P_2P_1}}{|\overrightarrow{P_2P_1}|} + F\frac{\overrightarrow{P_2P_3}}{|\overrightarrow{P_2P_3}|}$$
$$F_3 = F\frac{\overrightarrow{P_3P_2}}{|\overrightarrow{P_3P_2}|} + F\frac{\overrightarrow{P_3P_4}}{|\overrightarrow{P_3P_4}|}$$

To summarize, the assist moment ($M_{ankle\_}$) of the exosuit is calculated by multiplying the geometric force at each point with the moment arm relative to the rotation axis. This can be expressed as follows:

$$M_{ankleo} = \overrightarrow{OP_0} \times F_0 + \overrightarrow{OP_1} \times F_1 + \overrightarrow{OP_2} \times F_2 + \overrightarrow{OP_3} \times F_3$$
$$M_{ankleo}(x) = \frac{F(p4z\sigma3 + p3y\sigma1)}{\sqrt{\sigma1^2+\sigma2^2+\sigma3^2}}$$
$$M_{ankleo}(y) = -\frac{F(p4z\sigma2 + p3x\sigma1)}{\sqrt{\sigma1^2+\sigma2^2+\sigma3^2}}$$
$$M_{ankleo}(z) = -\frac{F(p3y\sigma2 - p3x\sigma3)}{\sqrt{\sigma1^2+\sigma2^2+\sigma3^2}}$$

Where,

$$\sigma1 = p4z + p3x\sin(flexion) - p3z\cos(flexion)\cos(inversion) - p3y\cos(flexion)\sin(inversion)$$
$$\sigma2 = p3x\cos(adduction)\cos(flexion) - p3x - p3y\cos(inversion)\sin(adduction) + p3z\sin(adduction)\sin(inversion) + p3z\cos(adduction)\cos(inversion)\sin(flexion) + p3y\cos(adduction)\sin(flexion)\sin(inversion)$$
$$\sigma3 = p3y\cos(adduction)\cos(inversion) - p3y + p3x\cos(flexion)\sin(adduction) - p3z\cos(adduction)\sin(inversion) + p3z\cos(inversion)\sin(adduction)\sin(flexion) + p3y\sin(adduction)\sin(flexion)\sin(inversion)$$

### 2) Calculation of the assistance moment considering the ankle joint axes

However, the rotation of the ankle joint exhibits anisotropy, occurring along two distinct axes: the subtalar joint axis and the talocrural joint axis. Therefore, the moment generated by an exosuit must be adjusted along both axes. We independently calculated the rotational moments of the exosuit to the foot along these two axes. Then, by summing these moments together, we determined the overall assisting moment provided by the exosuit to the ankle joint.

To calculate the moment affecting the talocrural joint, we rotated the original coordinate system $O$ to the coordinate system $O''$, where the y-axis represents the distance from the talocrural joint axis. By determining the moment from the talocrural joint according to the moment of the exosuit force on the y-axis in the current coordinate system, we calculated the moment on the talocrural joint.

First, we solved the coordinates of the exosuit action point in the $O''$ coordinate system through coordinate system transformation. It was known that the rotation axis of the talocrural joint generally forms an angle of $angle1 = 6°$ with the frontal plane, and forms an angle of $angle2 = 10°$ with the horizontal plane. We obtained the rotation matrix and the coordinates of the point of action after rotation:s:

$$P_{italocrural} = R(\beta_z)R(\beta_x)P_i, i = 0,1,2,3,4$$
$$R(\beta_x) = [1,0,0; \cos(angle1), \sin(angle1), 0; 0, -\sin(angle1), \cos(angle1)]$$
$$R(\beta_z) = [\cos(angle12), \sin(angle12), 0; -\sin(angle12), \cos(angle12), 0; 0,0,1]$$

Where,

$$\cos(angle12) = \frac{\sqrt{\tan^2(angle2)+1}}{\sqrt{\tan^2(angle2)+\tan^2(angle2)+1}},$$
$$\sin(angle12) = -\frac{\tan(angle2)}{\sqrt{\tan^2(angle2)+\tan^2(angle2)+1}}$$

Next, we substituted $P_{italocrural}$ into the formula (1-5) to solve the moment $M_{italocrural\_}$ under the coordinate system $O''$.

Finally, we projected the moment component on the Y-axis onto the coordinate system $O$:

$$M_{talocrural} = R(\varphi_x)'R(\varphi_z)'M_{italocrural\_}[0,1,0]'$$

Similarly, we solved for the moment on the subtalar joint. we rotated the original coordinate system $O$ to a new coordinate system $O'$. In this new system, the X-axis coincides with the axis of the subtalar joint. At this point, the moment generated by any exosuit force acting solely on the X-axis o is equivalent to the assistive moment of the subtalar joint.

First, we solved the coordinates of the exosuit action point in the $O'$ coordinate system through coordinate system transformation. It was known that the rotation axis of the subtalar joint forms an angle of $angle3 = 16°$ with the sagittal plane and an angle of $angle4 = 42°$ with the horizontal plane. Using geometric relations, we obtained the rotation matrix and the coordinates of the point of action after rotation:

$$P_{subtalari} = R(\varphi_y)R(\varphi_z)P_i, i = 0,1,2,3,4$$

$$R(\varphi_z) = [\cos(angle3), \sin(angle3), 0; -\sin(angle3), \cos(angle3), 0; 0,0,1]$$

$$R(\varphi_y) = [\cos(angle34), 0, -\sin(angle34); 0,1,0; \sin(angle34), 0, \cos(angle34)]$$

where,

$$\cos(angle34) = \frac{\sqrt{\tan^2(angle3)+1}}{\sqrt{\tan^2(angle3)+\tan^2(angle4)+1}},$$
$$\sin(angle34) = -\frac{\tan(angle4)}{\sqrt{\tan^2(angle3)+\tan^2(angle4)+1}}$$

Next, we substituted the coordinates of $P_{isubtalar}$ into equation (1-5) to solve for the force ($F_{isubtalar\_}$)and rotational moment ($M_{subtalar\_}$) of the four points on foot under the $O'$ coordinate system.

$$M_{subtalar\_} = \overrightarrow{OP_{0subtalar}} \times F_{0subtalar} + \overrightarrow{OP_{1subtalar}} \times F_{1subtalar} + \overrightarrow{OP_{2subtalar}} \times F_{2subtalar} + \overrightarrow{OP_{3subtalar}} \times F_{3subtalar}$$

Finally, we projected the moment component of the rotational moment ($M_{subtalar\_}$) on the X-axis under the $O'$coordinate system onto the three-dimensional coordinate axes of the coordinate system $O$:

$$M_{subtalar} = R(\varphi_z)'R(\varphi_y)'M_{subtalar\_}[1,0,0]'$$

where, $R(\varphi_z)'$and $R(\varphi_y)'$ are the inverses of $R(\varphi_z)$ and $R(\varphi_y)$.

In summary, the comprehensive moment of the exosuit to the ankle joint is obtained:

$$M_{ankle} = [M_x, M_y, M_z] = M_{subtalar} + M_{talocrural}$$

Where,

$M_x$ = (F cos(angle3) cos(angle4) √(−(cos(angle3))^2 (cos(angle4))^2 + (cos(angle3))^2 + (cos(angle4))^2 ) ((p3x)^2 cos(angle4) sin(angle3) sin(flexion) + (p3y)^2 cos(angle3) cos(angle4) cos(flexion) sin(inversion) − p3x p3y cos(angle3) cos(angle4) sin(flexion) + ((p3x)^2 (cos(angle3))^2 cos(flexion) sin(adduction) sin(angle4))/(|cos(angle3)|) + ((p3y)^2 (cos(angle3))^2 sin(adduction) sin(angle4) cos(inversion))/(|cos(angle3)|) − p3y p4z cos(adduction) cos(angle3) cos(angle4) cos(inversion) + p3x p4z cos(adduction) cos(angle4) cos(flexion) sin(angle3) − p3x p4z cos(angle3) cos(angle4) cos(flexion) sin(adduction) + p3y p3z cos(angle3) cos(angle4) cos(flexion) cos(inversion) + p3z p4z cos(adduction) cos(angle3) cos(angle4) sin(inversion) − p3x p3z cos(angle4) cos(flexion) sin(angle3) cos(inversion) − p3y p4z cos(angle4) sin(adduction) sin(angle3) cos(inversion) − p3x p3y cos(angle4) cos(flexion) sin(angle3) sin(inversion) + p3z p4z cos(angle4) sin(adduction) sin(angle3) sin(inversion) − ((p3y)^2 cos(adduction) (cos(angle3))^2 sin(angle4) sin(flexion) sin(inversion))/(|cos(angle3)|) − (p3x p3y cos(adduction) (cos(angle3))^2 cos(flexion) sin(angle4))/(|cos(angle3)|) + p3z p4z cos(adduction) cos(angle4) sin(angle3) cos(inversion) sin(flexion) − p3z p4z cos(angle3) cos(angle4) sin(adduction) cos(inversion) sin(flexion) + (p3x p3y cos(adduction) (cos(angle3))^2 sin(angle4) cos(inversion))/(|cos(angle3)|) + p3y p4z cos(adduction) cos(angle4) sin(angle3) sin(flexion) sin(inversion) − p3y p4z cos(angle3) cos(angle4) sin(adduction) sin(flexion) sin(inversion) − (p3x p3z cos(adduction) (cos(angle3))^2 sin(angle4) sin(inversion))/(|cos(angle3)|) − (p3y p3z (cos(angle3))^2 sin(adduction) sin(angle4) sin(inversion))/(|cos(angle3)|) − (p3y p3z cos(adduction) (cos(angle3))^2 sin(angle4) cos(inversion) sin(flexion))/(|cos(angle3)|) + (p3x p3z (cos(angle3))^2 sin(adduction) sin(angle4) cos(inversion) sin(flexion))/(|cos(angle3)|) + (p3x p3y (cos(angle3))^2 sin(adduction) sin(angle4) sin(flexion) sin(inversion))/(|cos(angle3)|)))/(((sin(angle3))^2 (sin(angle4))^2 − 1) √((p4z cos(angle4) + p3x cos(angle4) sin(flexion) − p3z cos(angle4) cos(flexion) cos(inversion) − p3y cos(angle4) cos(flexion) sin(inversion) − p3x |cos(angle3)| cos(angle3) sin(angle4) − p3y |cos(angle3)| sin(angle3) sin(angle4) + p3x |cos(angle3)| cos(adduction) cos(angle3) cos(flexion) sin(angle4) + p3y |cos(angle3)| cos(adduction) sin(angle3) sin(angle4) cos(inversion) − p3y |cos(angle3)| cos(angle3) sin(adduction) sin(angle4) cos(inversion) + p3x |cos(angle3)| cos(flexion) sin(adduction) sin(angle3) sin(angle4) −

p3z |cos(angle3)| cos(adduction) sin(angle3) sin(angle4) sin(inversion) +
p3z |cos(angle3)| cos(angle3) sin(adduction) sin(angle4) sin(inversion) +
p3z |cos(angle3)| cos(adduction) cos(angle3) sin(angle4) cos(inversion) sin(flexion) +
p3y |cos(angle3)| cos(adduction) cos(angle3) sin(angle4) sin(flexion) sin(inversion) +
p3z |cos(angle3)| sin(adduction) sin(angle3) sin(angle4) cos(inversion) sin(flexion) +
p3y |cos(angle3)| sin(adduction) sin(angle3) sin(angle4) sin(flexion) sin(inversion))^2 + (cos(angle3))^2 σ1 + (cos(angle4))^2 σ1 +
(p3y cos(adduction) cos(angle4) sin(angle3) cos(inversion) − p3y cos(angle4) sin(angle3) − p4z |cos(angle3)| sin(angle4) −
p3x |cos(angle3)| sin(angle4) sin(flexion) − p3x cos(angle3) cos(angle4) − p3y cos(angle3) cos(angle4) sin(adduction) cos(inversion) +
p3x cos(angle4) cos(flexion) sin(adduction) sin(angle3) − p3z cos(adduction) cos(angle4) sin(angle3) sin(inversion) +
p3z cos(angle3) cos(angle4) sin(adduction) sin(inversion) + p3z |cos(angle3)| cos(flexion) sin(angle4) cos(inversion) +
p3y |cos(angle3)| cos(flexion) sin(angle4) sin(inversion) + p3x cos(adduction) cos(angle3) cos(angle4) cos(flexion) +
p3y cos(angle4) sin(adduction) sin(angle3) sin(flexion) sin(inversion) +
p3z cos(adduction) cos(angle3) cos(angle4) cos(inversion) sin(flexion) +
p3y cos(adduction) cos(angle3) cos(angle4) sin(flexion) sin(inversion) +
p3z cos(angle4) sin(adduction) sin(angle3) cos(inversion) sin(flexion))^2 − (cos(angle3))^2 (cos(angle4))^2 σ1 )) +
(2 F (cos(angle2))^2 √(−(cos(angle1))^2 (cos(angle2))^2 + (cos(angle1))^2 +
(cos(angle2))^2 ) ((p3x)^2 (cos(angle1))^2 sin(angle1) sin(flexion) − (p3y)^2 (cos(angle1))^3 sin(adduction) cos(inversion) +
(p3y)^2 |cos(angle2)| cos(flexion) sin(inversion) − p3x p3y |cos(angle2)| sin(flexion) + (p3x)^2 cos(angle1) cos(flexion) sin(adduction) +
(p3y)^2 cos(angle1) sin(adduction) cos(inversion) − (p3x)^2 (cos(angle1))^3 cos(flexion) sin(adduction) −
p3x p3y cos(adduction) (cos(angle1))^3 cos(inversion) − p3y p4z |cos(angle2)| cos(adduction) cos(inversion) −
p3x p4z |cos(angle2)| cos(flexion) sin(adduction) + p3y p3z |cos(angle2)| cos(flexion) cos(inversion) +
p3x p3z cos(adduction) (cos(angle1))^3 sin(inversion) + p3z p4z |cos(angle2)| cos(adduction) sin(inversion) +
p3y p3z (cos(angle1))^3 sin(adduction) sin(inversion) − (p3y)^2 |cos(angle2)| (cos(angle1))^2 cos(flexion) sin(inversion) +
p3x p3y |cos(angle2)| (cos(angle1))^2 sin(flexion) − (p3y)^2 cos(adduction) cos(angle1) sin(flexion) sin(inversion) −
p3x p3y cos(adduction) cos(angle1) cos(flexion) + p3x p3y cos(adduction) cos(angle1) cos(inversion) −
p3x p3z cos(adduction) cos(angle1) sin(inversion) − p3y p3z cos(angle1) sin(adduction) sin(inversion) +
(p3y)^2 cos(adduction) (cos(angle1))^3 sin(flexion) sin(inversion) + p3x p3y cos(adduction) (cos(angle1))^3 cos(flexion) +
p3y p4z |cos(angle2)| cos(adduction) (cos(angle1))^2 cos(inversion) + p3x p4z |cos(angle2)| (cos(angle1))^2 cos(flexion) sin(adduction) −
p3y p3z |cos(angle2)| (cos(angle1))^2 cos(flexion) cos(inversion) − p3z p4z |cos(angle2)| cos(adduction) (cos(angle1))^2 sin(inversion) −
p3y p3z cos(adduction) cos(angle1) cos(inversion) sin(flexion) + p3x p3z cos(angle1) sin(adduction) cos(inversion) sin(flexion) +
p3x p3y cos(angle1) sin(adduction) sin(flexion) sin(inversion) + p3x p4z cos(adduction) (cos(angle1))^2 cos(flexion) sin(angle1) +
p3y p3z cos(adduction) (cos(angle1))^3 cos(inversion) sin(flexion) − p3x p3z (cos(angle1))^2 cos(flexion) sin(angle1) cos(inversion) −
p3y p4z (cos(angle1))^2 sin(adduction) sin(angle1) cos(inversion) − p3x p3z (cos(angle1))^3 sin(adduction) cos(inversion) sin(flexion) −
p3x p3y (cos(angle1))^2 cos(flexion) sin(angle1) sin(inversion) − p3z p4z |cos(angle2)| sin(adduction) cos(inversion) sin(flexion) +
p3z p4z (cos(angle1))^2 sin(adduction) sin(angle1) sin(inversion) − p3x p3y (cos(angle1))^3 sin(adduction) sin(flexion) sin(inversion) −
p3y p4z |cos(angle2)| sin(adduction) sin(flexion) sin(inversion) +
p3z p4z |cos(angle2)| (cos(angle1))^2 sin(adduction) cos(inversion) sin(flexion) +
p3y p4z |cos(angle2)| (cos(angle1))^2 sin(adduction) sin(flexion) sin(inversion) +
p3z p4z cos(adduction) (cos(angle1))^2 sin(angle1) cos(inversion) sin(flexion) +
p3y p4z cos(adduction) (cos(angle1))^2 sin(angle1) sin(flexion) sin(inversion)))/(|cos(angle2)| ((sin(angle1))^2 (sin(angle2))^2 −
1) √(4 (cos(angle1))^2 σ2 + 4 (cos(angle2))^2 σ2 + (2 p3y cos(adduction) (cos(angle1))^2 cos(inversion) − 2 p3y (cos(angle1))^2 −
2 p3x |cos(angle2)| sin(angle1) − 2 p3x cos(angle1) sin(angle1) sin(flexion) − p4z sin(2 angle1) +
2 p3x (cos(angle1))^2 cos(flexion) sin(adduction) − 2 p3z cos(adduction) (cos(angle1))^2 sin(inversion) +
2 p3z cos(angle1) cos(flexion) sin(angle1) cos(inversion) + 2 p3y cos(angle1) cos(flexion) sin(angle1) sin(inversion) +
2 p3x |cos(angle2)| cos(adduction) cos(flexion) sin(angle1) − 2 p3y |cos(angle2)| sin(adduction) sin(angle1) cos(inversion) +
2 p3z (cos(angle1))^2 sin(adduction) cos(inversion) sin(flexion) + 2 p3z |cos(angle2)| sin(adduction) sin(angle1) sin(inversion) +
2 p3y (cos(angle1))^2 sin(adduction) sin(flexion) sin(inversion) +
2 p3z |cos(angle2)| cos(adduction) sin(angle1) cos(inversion) sin(flexion) +
2 p3y |cos(angle2)| cos(adduction) sin(angle1) sin(flexion) sin(inversion))^2 + (2 p3x cos(angle1) − 2 p4z |cos(angle2)| +
2 p4z |cos(angle2)| (cos(angle1))^2 − p3y |cos(angle2)| sin(2 angle1) − 2 p3x |cos(angle2)| sin(flexion) −
2 p3x cos(adduction) cos(angle1) cos(flexion) + 2 p3y cos(angle1) sin(adduction) cos(inversion) −
2 p3z cos(angle1) sin(adduction) sin(inversion) + 2 p3z |cos(angle2)| cos(flexion) cos(inversion) +
2 p3y |cos(angle2)| cos(flexion) sin(inversion) + 2 p3x |cos(angle2)| (cos(angle1))^2 sin(flexion) −
2 p3z cos(adduction) cos(angle1) cos(inversion) sin(flexion) − 2 p3y cos(adduction) cos(angle1) sin(flexion) sin(inversion) −
2 p3z |cos(angle2)| (cos(angle1))^2 cos(flexion) cos(inversion) − 2 p3y |cos(angle2)| (cos(angle1))^2 cos(flexion) sin(inversion) +
2 p3y |cos(angle2)| cos(adduction) cos(angle1) sin(angle1) cos(inversion) +
2 p3x |cos(angle2)| cos(angle1) cos(flexion) sin(adduction) sin(angle1) −
2 p3z |cos(angle2)| cos(adduction) cos(angle1) sin(angle1) sin(inversion) +
2 p3z |cos(angle2)| cos(angle1) sin(adduction) sin(angle1) cos(inversion) sin(flexion) +
2 p3y |cos(angle2)| cos(angle1) sin(adduction) sin(angle1) sin(flexion) sin(inversion))^2 − 4 (cos(angle1))^2 (cos(angle2))^2 σ2 ))

where
$\sigma_1$ = (p3y cos($\mathrm{angle}_3$) − p3x sin($\mathrm{angle}_3$) − p3y cos(adduction) cos($\mathrm{angle}_3$) cos(inversion) + p3x cos(adduction) cos(flexion) sin($\mathrm{angle}_3$) −
p3x cos($\mathrm{angle}_3$) cos(flexion) sin(adduction) + p3z cos(adduction) cos($\mathrm{angle}_3$) sin(inversion) −
p3y sin(adduction) sin($\mathrm{angle}_3$) cos(inversion) + p3z sin(adduction) sin($\mathrm{angle}_3$) sin(inversion) +
p3z cos(adduction) sin($\mathrm{angle}_3$) cos(inversion) sin(flexion) − p3z cos($\mathrm{angle}_3$) sin(adduction) cos(inversion) sin(flexion) +

p3y cos(adduction) sin($angle_3$) sin(flexion) sin(inversion) − p3y cos($angle_3$) sin(adduction) sin(flexion) sin(inversion))$^2$

$\sigma_2$ = (p4z cos($angle_1$) − p3y sin($angle_1$) + p3x cos($angle_1$) sin(flexion) − p3z cos($angle_1$) cos(flexion) cos(inversion) +
p3y cos(adduction) sin($angle_1$) cos(inversion) + p3x cos(flexion) sin(adduction) sin($angle_1$) − p3y cos($angle_1$) cos(flexion) sin(inversion) −
p3z cos(adduction) sin($angle_1$) sin(inversion) + p3z sin(adduction) sin($angle_1$) cos(inversion) sin(flexion) +
p3y sin(adduction) sin($angle_1$) sin(flexion) sin(inversion))$^2$

$M_y$ = (2 $F$(cos(angle1))^2 √(−(cos(angle1))^2(cos(angle2))^2 + (cos(angle1))^2 +
(cos(angle2))^2 ) ((p3x)^2(cos(angle1))^2 sin(flexion) − p3x p3z(cos(angle1))^2 cos(flexion) cos(inversion) −
p3y p4z(cos(angle1))^2 sin(adduction) cos(inversion) − p3x p3y(cos(angle1))^2 cos(flexion) sin(inversion) +
p3z p4z(cos(angle1))^2 sin(adduction) sin(inversion) + (p3x)^2 cos(angle1) cos(flexion) sin(adduction) sin(angle1) +
(p3y)^2 cos(angle1) sin(adduction) sin(angle1) cos(inversion) + p3x p4z cos(adduction)(cos(angle1))^2 cos(flexion) −
(p3y)^2 cos(adduction) cos(angle1) sin(angle1) sin(flexion) sin(inversion) +
((p3y)^2(cos(angle2))^2 cos(flexion) sin(angle1) sin(inversion))/(|cos(angle2)|) −
p3x p3y cos(adduction) cos(angle1) cos(flexion) sin(angle1) + p3x p3y cos(adduction) cos(angle1) sin(angle1) cos(inversion) −
p3x p3z cos(adduction) cos(angle1) sin(angle1) sin(inversion) − p3y p3z cos(angle1) sin(adduction) sin(angle1) sin(inversion) −
(p3x p3y(cos(angle2))^2 sin(angle1) sin(flexion))/(|cos(angle2)|) + p3z p4z cos(adduction)(cos(angle1))^2 cos(inversion) sin(flexion) +
p3y p4z cos(adduction)(cos(angle1))^2 sin(flexion) sin(inversion) −
p3y p3z cos(adduction) cos(angle1) sin(angle1) cos(inversion) sin(flexion) −
(p3y p4z cos(adduction)(cos(angle2))^2 sin(angle1) cos(inversion))/(|cos(angle2)|) −
(p3x p4z(cos(angle2))^2 cos(flexion) sin(adduction) sin(angle1))/(|cos(angle2)|) +
p3x p3z cos(angle1) sin(adduction) sin(angle1) cos(inversion) sin(flexion) +
(p3y p3z(cos(angle2))^2 cos(flexion) sin(angle1) cos(inversion))/(|cos(angle2)|) +
(p3z p4z cos(adduction)(cos(angle2))^2 sin(angle1) sin(inversion))/(|cos(angle2)|) +
p3x p3y cos(angle1) sin(adduction) sin(angle1) sin(flexion) sin(inversion) −
(p3z p4z(cos(angle2))^2 sin(adduction) sin(angle1) cos(inversion) sin(flexion))/(|cos(angle2)|) −
(p3y p4z(cos(angle2))^2 sin(adduction) sin(angle1) sin(flexion) sin(inversion))/(|cos(angle2)|)))/(((sin(angle1))^2(sin(angle2))^2 −
1) √(4(cos(angle1))^2 $\sigma$2 + 4(cos(angle2))^2 $\sigma$2 + (2 p3y cos(adduction)(cos(angle1))^2 cos(inversion) − 2 p3y(cos(angle1))^2 −
2 p3x |cos(angle2)| sin(angle1) − 2 p3x cos(angle1) sin(angle1) sin(flexion) − p4z sin(2 angle1) +
2 p3x(cos(angle1))^2 cos(flexion) sin(adduction) − 2 p3z cos(adduction)(cos(angle1))^2 sin(inversion) +
2 p3z cos(angle1) cos(flexion) sin(angle1) cos(inversion) + 2 p3y cos(angle1) cos(flexion) sin(angle1) sin(inversion) +
2 p3x |cos(angle2)| cos(adduction) cos(flexion) sin(angle1) − 2 p3y |cos(angle2)| sin(adduction) sin(angle1) cos(inversion) +
2 p3z(cos(angle1))^2 sin(adduction) cos(inversion) sin(flexion) + 2 p3z |cos(angle2)| sin(adduction) sin(angle1) sin(inversion) +
2 p3y(cos(angle1))^2 sin(adduction) sin(flexion) sin(inversion) +
2 p3z |cos(angle2)| cos(adduction) sin(angle1) cos(inversion) sin(flexion) +
2 p3y |cos(angle2)| cos(adduction) sin(angle1) sin(flexion) sin(inversion))^2 + (2 p3x cos(angle1) − 2 p4z |cos(angle2)| +
2 p4z |cos(angle2)|(cos(angle1))^2 − p3y |cos(angle2)| sin(2 angle1) − 2 p3x |cos(angle2)| sin(flexion) −
2 p3x cos(adduction) cos(angle1) cos(flexion) + 2 p3y cos(angle1) sin(adduction) cos(inversion) −
2 p3z cos(angle1) sin(adduction) sin(inversion) + 2 p3z |cos(angle2)| cos(flexion) cos(inversion) +
2 p3y |cos(angle2)| cos(flexion) sin(inversion) + 2 p3x |cos(angle2)|(cos(angle1))^2 sin(flexion) −
2 p3z cos(adduction) cos(angle1) cos(inversion) sin(flexion) − 2 p3y cos(adduction) cos(angle1) sin(flexion) sin(inversion) −
2 p3z |cos(angle2)|(cos(angle1))^2 cos(flexion) cos(inversion) − 2 p3y |cos(angle2)|(cos(angle1))^2 cos(flexion) sin(inversion) +
2 p3y |cos(angle2)| cos(adduction) cos(angle1) sin(angle1) cos(inversion) +
2 p3x |cos(angle2)| cos(angle1) cos(flexion) sin(adduction) sin(angle1) −
2 p3z |cos(angle2)| cos(adduction) cos(angle1) sin(angle1) sin(inversion) +
2 p3z |cos(angle2)| cos(angle1) sin(adduction) sin(angle1) cos(inversion) sin(flexion) +
2 p3y |cos(angle2)| cos(angle1) sin(adduction) sin(angle1) sin(flexion) sin(inversion))^2 − 4(cos(angle1))^2(cos(angle2))^2 $\sigma$2 )) −
($F$ cos(angle4) √(−(cos(angle3))^2(cos(angle4))^2 + (cos(angle3))^2 +
(cos(angle4))^2 ) ((p3x)^2 |cos(angle3)|(cos(angle3))^2 cos(angle4) sin(flexion) − (p3x)^2 |cos(angle3)| cos(angle4) sin(flexion) −
(p3x)^2(cos(angle3))^2 cos(flexion) sin(adduction) sin(angle3) sin(angle4) −
(p3y)^2(cos(angle3))^2 sin(adduction) sin(angle3) sin(angle4) cos(inversion) −
p3x p4z |cos(angle3)| cos(adduction) cos(angle4) cos(flexion) + p3x p3z |cos(angle3)| cos(angle4) cos(flexion) cos(inversion) +
p3y p4z |cos(angle3)| cos(angle4) sin(adduction) cos(inversion) + p3x p3y |cos(angle3)| cos(angle4) cos(flexion) sin(inversion) −
p3z p4z |cos(angle3)| cos(angle4) sin(adduction) sin(inversion) −
p3x p3y |cos(angle3)|(cos(angle3))^2 cos(angle4) cos(flexion) sin(inversion) +
p3z p4z |cos(angle3)|(cos(angle3))^2 cos(angle4) sin(adduction) sin(inversion) −
(p3y)^2 |cos(angle3)| cos(angle3) cos(angle4) cos(flexion) sin(angle3) sin(inversion) +
(p3y)^2 cos(adduction)(cos(angle3))^2 sin(angle3) sin(angle4) sin(flexion) sin(inversion) +
p3x p3y cos(adduction)(cos(angle3))^2 cos(flexion) sin(angle3) sin(angle4) +
p3x p3y |cos(angle3)| cos(angle3) cos(angle4) sin(angle3) sin(flexion) −
p3z p4z |cos(angle3)| cos(adduction) cos(angle4) cos(inversion) sin(flexion) −
p3x p3y cos(adduction)(cos(angle3))^2 sin(angle3) sin(angle4) cos(inversion) −
p3y p4z |cos(angle3)| cos(adduction) cos(angle4) sin(flexion) sin(inversion) +
p3x p3z cos(adduction)(cos(angle3))^2 sin(angle3) sin(angle4) sin(inversion) +
p3y p3z(cos(angle3))^2 sin(adduction) sin(angle3) sin(angle4) sin(inversion) +

p3x p4z |cos(angle3)| cos(adduction)(cos(angle3))^2 cos(angle4) cos(flexion) −
p3x p3z |cos(angle3)|(cos(angle3))^2 cos(angle4) cos(flexion) cos(inversion) −
p3y p4z |cos(angle3)|(cos(angle3))^2 cos(angle4) sin(adduction) cos(inversion) +
p3y p4z |cos(angle3)| cos(adduction) cos(angle3) cos(angle4) sin(angle3) cos(inversion) +
p3x p4z |cos(angle3)| cos(angle3) cos(angle4) cos(flexion) sin(adduction) sin(angle3) −
p3y p3z |cos(angle3)| cos(angle3) cos(angle4) cos(flexion) sin(angle3) cos(inversion) −
p3z p4z |cos(angle3)| cos(adduction) cos(angle3) cos(angle4) sin(angle3) sin(inversion) +
p3y p3z cos(adduction)(cos(angle3))^2 sin(angle3) sin(angle4) cos(inversion) sin(flexion) −
p3x p3z(cos(angle3))^2 sin(adduction) sin(angle3) sin(angle4) cos(inversion) sin(flexion) −
p3x p3y(cos(angle3))^2 sin(adduction) sin(angle3) sin(angle4) sin(flexion) sin(inversion) +
p3z p4z |cos(angle3)| cos(adduction)(cos(angle3))^2 cos(angle4) cos(inversion) sin(flexion) +
p3y p4z |cos(angle3)| cos(adduction)(cos(angle3))^2 cos(angle4) sin(flexion) sin(inversion) +
p3z p4z |cos(angle3)| cos(angle3) cos(angle4) sin(adduction) sin(angle3) cos(inversion) sin(flexion) +
p3y p4z |cos(angle3)| cos(angle3) cos(angle4) sin(adduction) sin(angle3) sin(flexion) sin(inversion)))/
(|cos(angle3)| ((sin(angle3))^2(sin(angle4))^2 − 1) √((p4z cos(angle4) + p3x cos(angle4) sin(flexion) −
p3z cos(angle4) cos(flexion) cos(inversion) − p3y cos(angle4) cos(flexion) sin(inversion) − p3x |cos(angle3)| cos(angle3) sin(angle4) −
p3y |cos(angle3)| sin(angle3) sin(angle4) + p3x |cos(angle3)| cos(adduction) cos(angle3) cos(flexion) sin(angle4) +
p3y |cos(angle3)| cos(adduction) sin(angle3) sin(angle4) cos(inversion) −
p3y |cos(angle3)| cos(angle3) sin(adduction) sin(angle4) cos(inversion) +
p3x |cos(angle3)| cos(flexion) sin(adduction) sin(angle3) sin(angle4) −
p3z |cos(angle3)| cos(adduction) sin(angle3) sin(angle4) sin(inversion) +
p3z |cos(angle3)| cos(angle3) sin(adduction) sin(angle4) sin(inversion) +
p3z |cos(angle3)| cos(adduction) cos(angle3) sin(angle4) cos(inversion) sin(flexion) +
p3y |cos(angle3)| cos(adduction) cos(angle3) sin(angle4) sin(flexion) sin(inversion) +
p3z |cos(angle3)| sin(adduction) sin(angle3) sin(angle4) cos(inversion) sin(flexion) +
p3y |cos(angle3)| sin(adduction) sin(angle3) sin(angle4) sin(flexion) sin(inversion))^2 + (cos(angle3))^2 $\sigma1$ + (cos(angle4))^2 $\sigma1$ +
(p3y cos(adduction) cos(angle4) sin(angle3) cos(inversion) − p3y cos(angle4) sin(angle3) − p4z |cos(angle3)| sin(angle4) −
p3x |cos(angle3)| sin(angle4) sin(flexion) − p3x cos(angle3) cos(angle4) − p3y cos(angle3) cos(angle4) sin(adduction) cos(inversion) +
p3x cos(angle4) cos(flexion) sin(adduction) sin(angle3) − p3z cos(adduction) cos(angle4) sin(angle3) sin(inversion) +
p3z cos(angle3) cos(angle4) sin(adduction) sin(inversion) + p3z |cos(angle3)| cos(flexion) sin(angle4) cos(inversion) +
p3y |cos(angle3)| cos(flexion) sin(angle4) sin(inversion) + p3x cos(adduction) cos(angle3) cos(angle4) cos(flexion) +
p3y cos(angle4) sin(adduction) sin(angle3) sin(flexion) sin(inversion) +
p3z cos(adduction) cos(angle3) cos(angle4) cos(inversion) sin(flexion) +
p3y cos(adduction) cos(angle3) cos(angle4) sin(flexion) sin(inversion) +
p3z cos(angle4) sin(adduction) sin(angle3) cos(inversion) sin(flexion))^2 − (cos(angle3))^2(cos(angle4))^2 $\sigma1$ ))

where
σ1 = (p3y cos(angle3) − p3x sin(angle3) − p3y cos(adduction) cos(angle3) cos(inversion) + p3x cos(adduction) cos(flexion) sin(angle3) −
p3x cos(angle3) cos(flexion) sin(adduction) + p3z cos(adduction) cos(angle3) sin(inversion) −
p3y sin(adduction) sin(angle3) cos(inversion) + p3z sin(adduction) sin(angle3) sin(inversion) +
p3z cos(adduction) sin(angle3) cos(inversion) sin(flexion) − p3z cos(angle3) sin(adduction) cos(inversion) sin(flexion) +
p3y cos(adduction) sin(angle3) sin(flexion) sin(inversion) − p3y cos(angle3) sin(adduction) sin(flexion) sin(inversion))^2

$\sigma2$ = (p4z cos(angle1) − p3y sin(angle1) + p3x cos(angle1) sin(flexion) − p3z cos(angle1) cos(flexion) cos(inversion) +
p3y cos(adduction) sin(angle1) cos(inversion) + p3x cos(flexion) sin(adduction) sin(angle1) − p3y cos(angle1) cos(flexion) sin(inversion) −
p3z cos(adduction) sin(angle1) sin(inversion) + p3z sin(adduction) sin(angle1) cos(inversion) sin(flexion) +
p3y sin(adduction) sin(angle1) sin(flexion) sin(inversion))^2

$M_z$ = ($F$(cos(angle3))^2 √(−(cos(angle3))^2(cos(angle4))^2 + (cos(angle3))^2 +
(cos(angle4))^2 ) ((p3y)^2 |cos(angle3)| sin(adduction) cos(inversion) + (p3x)^2 |cos(angle3)| cos(flexion) sin(adduction) +
p3x p3y |cos(angle3)| cos(adduction) cos(inversion) − p3x p3z |cos(angle3)| cos(adduction) sin(inversion) −
(p3x)^2 |cos(angle3)|(cos(angle4))^2 cos(flexion) sin(adduction) − p3y p3z |cos(angle3)| sin(adduction) sin(inversion) −
(p3y)^2 |cos(angle3)|(cos(angle4))^2 sin(adduction) cos(inversion) + (p3x)^2 cos(angle4) sin(angle3) sin(angle4) sin(flexion) −
(p3y)^2 |cos(angle3)| cos(adduction) sin(flexion) sin(inversion) − p3x p3y |cos(angle3)| cos(adduction) cos(flexion) +
(p3y)^2 |cos(angle3)| cos(adduction)(cos(angle4))^2 sin(flexion) sin(inversion) +
p3x p3y |cos(angle3)| cos(adduction)(cos(angle4))^2 cos(flexion) − p3x p3y |cos(angle3)| cos(adduction)(cos(angle4))^2 cos(inversion) +
p3x p3z |cos(angle3)| cos(adduction)(cos(angle4))^2 sin(inversion) +
(p3y)^2 cos(angle3) cos(angle4) cos(flexion) sin(angle4) sin(inversion) +
p3y p3z |cos(angle3)|(cos(angle4))^2 sin(adduction) sin(inversion) − p3x p3y cos(angle3) cos(angle4) sin(angle4) sin(flexion) −
p3y p3z |cos(angle3)| cos(adduction) cos(inversion) sin(flexion) + p3x p3z |cos(angle3)| sin(adduction) cos(inversion) sin(flexion) +
p3x p3y |cos(angle3)| sin(adduction) sin(flexion) sin(inversion) +
p3y p3z |cos(angle3)| cos(adduction)(cos(angle4))^2 cos(inversion) sin(flexion) −
p3x p3z |cos(angle3)|(cos(angle4))^2 sin(adduction) cos(inversion) sin(flexion) −
p3x p3y |cos(angle3)|(cos(angle4))^2 sin(adduction) sin(flexion) sin(inversion) −
p3y p4z cos(adduction) cos(angle3) cos(angle4) sin(angle4) cos(inversion) +
p3x p4z cos(adduction) cos(angle4) cos(flexion) sin(angle3) sin(angle4) −

p3x p4z cos(angle3) cos(angle4) cos(flexion) sin(adduction) sin(angle4) +
p3y p3z cos(angle3) cos(angle4) cos(flexion) sin(angle4) cos(inversion) +
p3z p4z cos(adduction) cos(angle3) cos(angle4) sin(angle4) sin(inversion) −
p3x p3z cos(angle4) cos(flexion) sin(angle3) sin(angle4) cos(inversion) −
p3y p4z cos(angle4) sin(adduction) sin(angle3) sin(angle4) cos(inversion) −
p3x p3y cos(angle4) cos(flexion) sin(angle3) sin(angle4) sin(inversion) +
p3z p4z cos(angle4) sin(adduction) sin(angle3) sin(angle4) sin(inversion) +
p3z p4z cos(adduction) cos(angle4) sin(angle3) sin(angle4) cos(inversion) sin(flexion) −
p3z p4z cos(angle3) cos(angle4) sin(adduction) sin(angle4) cos(inversion) sin(flexion) +
p3y p4z cos(adduction) cos(angle4) sin(angle3) sin(angle4) sin(flexion) sin(inversion) −
p3y p4z cos(angle3) cos(angle4) sin(adduction) sin(angle4) sin(flexion) sin(inversion)))/(|cos(angle3)| ((sin(angle3))^2(sin(angle4))^2 −
1) √((p4z cos(angle4) + p3x cos(angle4) sin(flexion) − p3z cos(angle4) cos(flexion) cos(inversion) −
p3y cos(angle4) cos(flexion) sin(inversion) − p3x |cos(angle3)| cos(angle3) sin(angle4) − p3y |cos(angle3)| sin(angle3) sin(angle4) +
p3x |cos(angle3)| cos(adduction) cos(angle3) cos(flexion) sin(angle4) +
p3y |cos(angle3)| cos(adduction) sin(angle3) sin(angle4) cos(inversion) −
p3y |cos(angle3)| cos(angle3) sin(adduction) sin(angle4) cos(inversion) +
p3x |cos(angle3)| cos(flexion) sin(adduction) sin(angle3) sin(angle4) −
p3z |cos(angle3)| cos(adduction) sin(angle3) sin(angle4) sin(inversion) +
p3z |cos(angle3)| cos(angle3) sin(adduction) sin(angle4) sin(inversion) +
p3z |cos(angle3)| cos(adduction) cos(angle3) sin(angle4) cos(inversion) sin(flexion) +
p3y |cos(angle3)| cos(adduction) cos(angle3) sin(angle4) sin(flexion) sin(inversion) +
p3z |cos(angle3)| sin(adduction) sin(angle3) sin(angle4) cos(inversion) sin(flexion) +
p3y |cos(angle3)| sin(adduction) sin(angle3) sin(angle4) sin(flexion) sin(inversion))^2 + (cos(angle3))^2 σ1 + (cos(angle4))^2 σ1 +
(p3y cos(adduction) cos(angle4) sin(angle3) cos(inversion) − p3y cos(angle4) sin(angle3) − p4z |cos(angle3)| sin(angle4) −
p3x |cos(angle3)| sin(angle4) sin(flexion) − p3x cos(angle3) cos(angle4) − p3y cos(angle3) cos(angle4) sin(adduction) cos(inversion) +
p3x cos(angle4) cos(flexion) sin(adduction) sin(angle3) − p3z cos(adduction) cos(angle4) sin(angle3) sin(inversion) +
p3z cos(angle3) cos(angle4) sin(adduction) sin(inversion) + p3z |cos(angle3)| cos(flexion) sin(angle4) cos(inversion) +
p3y |cos(angle3)| cos(flexion) sin(angle4) sin(inversion) + p3x cos(adduction) cos(angle3) cos(angle4) cos(flexion) +
p3y cos(angle4) sin(adduction) sin(angle3) sin(flexion) sin(inversion) +
p3z cos(adduction) cos(angle3) cos(angle4) cos(inversion) sin(flexion) +
p3y cos(adduction) cos(angle3) cos(angle4) sin(flexion) sin(inversion) +
p3z cos(angle4) sin(adduction) sin(angle3) cos(inversion) sin(flexion))^2 − (cos(angle3))^2(cos(angle4))^2 σ1 )) +
(2 F cos(angle1) √(−(cos(angle1))^2(cos(angle2))^2 + (cos(angle1))^2 +
(cos(angle2))^2 ) ((p3y)^2(cos(angle2))^2 cos(flexion) sin(inversion) − p3x p3y(cos(angle2))^2 sin(flexion) −
p3y p4z cos(adduction)(cos(angle2))^2 cos(inversion) − p3x p4z(cos(angle2))^2 cos(flexion) sin(adduction) +
p3y p3z(cos(angle2))^2 cos(flexion) cos(inversion) + p3z p4z cos(adduction)(cos(angle2))^2 sin(inversion) −
(p3x)^2 |cos(angle2)|(cos(angle1))^3 cos(flexion) sin(adduction) − (p3y)^2 |cos(angle2)|(cos(angle1))^3 sin(adduction) cos(inversion) +
(p3x)^2 |cos(angle2)|(cos(angle1))^2 sin(angle1) sin(flexion) − (p3y)^2(cos(angle1))^2(cos(angle2))^2 cos(flexion) sin(inversion) +
p3x p3y(cos(angle1))^2(cos(angle2))^2 sin(flexion) + (p3x)^2 |cos(angle2)| cos(angle1) cos(flexion) sin(adduction) +
(p3y)^2 |cos(angle2)| cos(angle1) sin(adduction) cos(inversion) +
(p3y)^2 |cos(angle2)| cos(adduction)(cos(angle1))^3 sin(flexion) sin(inversion) +
p3x p3y |cos(angle2)| cos(adduction)(cos(angle1))^3 cos(flexion) +
p3y p4z cos(adduction)(cos(angle1))^2(cos(angle2))^2 cos(inversion) +
p3x p4z(cos(angle1))^2(cos(angle2))^2 cos(flexion) sin(adduction) − p3x p3y |cos(angle2)| cos(adduction)(cos(angle1))^3 cos(inversion) −
p3y p3z(cos(angle1))^2(cos(angle2))^2 cos(flexion) cos(inversion) −
p3z p4z cos(adduction)(cos(angle1))^2(cos(angle2))^2 sin(inversion) +
p3x p3z |cos(angle2)| cos(adduction)(cos(angle1))^3 sin(inversion) + p3y p3z |cos(angle2)|(cos(angle1))^3 sin(adduction) sin(inversion) −
(p3y)^2 |cos(angle2)| cos(adduction) cos(angle1) sin(flexion) sin(inversion) −
p3x p3y |cos(angle2)| cos(adduction) cos(angle1) cos(flexion) + p3x p3y |cos(angle2)| cos(adduction) cos(angle1) cos(inversion) −
p3x p3z |cos(angle2)| cos(adduction) cos(angle1) sin(inversion) − p3y p3z |cos(angle2)| cos(angle1) sin(adduction) sin(inversion) −
p3z p4z(cos(angle2))^2 sin(adduction) cos(inversion) sin(flexion) − p3y p4z(cos(angle2))^2 sin(adduction) sin(flexion) sin(inversion) +
p3y p3z |cos(angle2)| cos(adduction)(cos(angle1))^3 cos(inversion) sin(flexion) −
p3x p3z |cos(angle2)|(cos(angle1))^2 cos(flexion) sin(angle1) cos(inversion) −
p3y p4z |cos(angle2)|(cos(angle1))^2 sin(adduction) sin(angle1) cos(inversion) +
p3z p4z(cos(angle1))^2(cos(angle2))^2 sin(adduction) cos(inversion) sin(flexion) −
p3x p3z |cos(angle2)|(cos(angle1))^3 sin(adduction) cos(inversion) sin(flexion) −
p3x p3y |cos(angle2)|(cos(angle1))^2 cos(flexion) sin(angle1) sin(inversion) +
p3z p4z |cos(angle2)|(cos(angle1))^2 sin(adduction) sin(angle1) sin(inversion) +
p3y p4z(cos(angle1))^2(cos(angle2))^2 sin(adduction) sin(flexion) sin(inversion) −
p3x p3y |cos(angle2)|(cos(angle1))^3 sin(adduction) sin(flexion) sin(inversion) −
p3y p3z |cos(angle2)| cos(adduction) cos(angle1) cos(inversion) sin(flexion) +
p3x p3z |cos(angle2)| cos(angle1) sin(adduction) cos(inversion) sin(flexion) +
p3x p3y |cos(angle2)| cos(angle1) sin(adduction) sin(flexion) sin(inversion) +
p3x p4z |cos(angle2)| cos(adduction)(cos(angle1))^2 cos(flexion) sin(angle1) +
p3z p4z |cos(angle2)| cos(adduction)(cos(angle1))^2 sin(angle1) cos(inversion) sin(flexion) +
p3y p4z |cos(angle2)| cos(adduction)(cos(angle1))^2 sin(angle1) sin(flexion) sin(inversion)))/

(|cos(angle2)| ((sin(angle1))^2(sin(angle2))^2 − 1) √(4(cos(angle1))^2 σ2 + 4(cos(angle2))^2 σ2 +
(2 p3y cos(adduction)(cos(angle1))^2 cos(inversion) − 2 p3y(cos(angle1))^2 − 2 p3x |cos(angle2)| sin(angle1) −
2 p3x cos(angle1) sin(angle1) sin(flexion) − p4z sin(2 angle1) + 2 p3x(cos(angle1))^2 cos(flexion) sin(adduction) −
2 p3z cos(adduction)(cos(angle1))^2 sin(inversion) + 2 p3z cos(angle1) cos(flexion) sin(angle1) cos(inversion) +
2 p3y cos(angle1) cos(flexion) sin(angle1) sin(inversion) + 2 p3x |cos(angle2)| cos(adduction) cos(flexion) sin(angle1) −
2 p3y |cos(angle2)| sin(adduction) sin(angle1) cos(inversion) + 2 p3z(cos(angle1))^2 sin(adduction) cos(inversion) sin(flexion) +
2 p3z |cos(angle2)| sin(adduction) sin(angle1) sin(inversion) + 2 p3y(cos(angle1))^2 sin(adduction) sin(flexion) sin(inversion) +
2 p3z |cos(angle2)| cos(adduction) sin(angle1) cos(inversion) sin(flexion) +
2 p3y |cos(angle2)| cos(adduction) sin(angle1) sin(flexion) sin(inversion))^2 + (2 p3x cos(angle1) − 2 p4z |cos(angle2)| +
2 p4z |cos(angle2)|(cos(angle1))^2 − p3y |cos(angle2)| sin(2 angle1) − 2 p3x |cos(angle2)| sin(flexion) −
2 p3x cos(adduction) cos(angle1) cos(flexion) + 2 p3y cos(angle1) sin(adduction) cos(inversion) −
2 p3z cos(angle1) sin(adduction) sin(inversion) + 2 p3z |cos(angle2)| cos(flexion) cos(inversion) +
2 p3y |cos(angle2)| cos(flexion) sin(inversion) + 2 p3x |cos(angle2)|(cos(angle1))^2 sin(flexion) −
2 p3z cos(adduction) cos(angle1) cos(inversion) sin(flexion) − 2 p3y cos(adduction) cos(angle1) sin(flexion) sin(inversion) −
2 p3z |cos(angle2)|(cos(angle1))^2 cos(flexion) cos(inversion) − 2 p3y |cos(angle2)|(cos(angle1))^2 cos(flexion) sin(inversion) +
2 p3y |cos(angle2)| cos(adduction) cos(angle1) sin(angle1) cos(inversion) +
2 p3x |cos(angle2)| cos(angle1) cos(flexion) sin(adduction) sin(angle1) −
2 p3z |cos(angle2)| cos(adduction) cos(angle1) sin(angle1) sin(inversion) +
2 p3z |cos(angle2)| cos(angle1) sin(adduction) sin(angle1) cos(inversion) sin(flexion) +
2 p3y |cos(angle2)| cos(angle1) sin(adduction) sin(angle1) sin(flexion) sin(inversion))^2 − 4(cos(angle1))^2(cos(angle2))^2 σ2 ))

where

σ1 = (p3y cos(angle3) − p3x sin(angle3) − p3y cos(adduction) cos(angle3) cos(inversion) + p3x cos(adduction) cos(flexion) sin(angle3) −
p3x cos(angle3) cos(flexion) sin(adduction) + p3z cos(adduction) cos(angle3) sin(inversion) −
p3y sin(adduction) sin(angle3) cos(inversion) + p3z sin(adduction) sin(angle3) sin(inversion) +
p3z cos(adduction) sin(angle3) cos(inversion) sin(flexion) − p3z cos(angle3) sin(adduction) cos(inversion) sin(flexion) +
p3y cos(adduction) sin(angle3) sin(flexion) sin(inversion) − p3y cos(angle3) sin(adduction) sin(flexion) sin(inversion))^2

σ2 = (p4z cos(angle1) − p3y sin(angle1) + p3x cos(angle1) sin(flexion) − p3z cos(angle1) cos(flexion) cos(inversion) +
p3y cos(adduction) sin(angle1) cos(inversion) + p3x cos(flexion) sin(adduction) sin(angle1) − p3y cos(angle1) cos(flexion) sin(inversion) −
p3z cos(adduction) sin(angle1) sin(inversion) + p3z sin(adduction) sin(angle1) cos(inversion) sin(flexion) +
p3y sin(adduction) sin(angle1) sin(flexion) sin(inversion)
